# Neurodevelopmental outcomes of children born to diabetic mothers in the Qatari population: a retrospective study

**DOI:** 10.1136/bmjpo-2025-004232

**Published:** 2026-05-22

**Authors:** Razan M Masri, Mohammad A A Bayoumi, Rasha I Amin, Hafsa O A Alsharif, Amna Musa, Ahmad S Mudrek, Basma A M R Selim, Prem Chandra, Samah Elshaar

**Affiliations:** 1 Department of Developmental Pediatrics, Hamad Medical Corporation, Doha, Qatar; 2 Neonatal Intensive Care Unit (NICU), Women’s Wellness and Research Center (WWRC), Hamad Medical Corporation, Doha, Qatar; 3 Department of Medical Education, Sidra Medicine, Doha, Qatar; 4 Department of Medical Education, Hamad Medical Corporation, Doha, Qatar; 5 Division of Critical Care Medicine, Department of Pediatrics, University of British Columbia, Vancouver, British Columbia, Canada; 6 West Bay Medicare (WBM), Doha, Qatar; 7 Medical Research Center, Hamad Medical Corporation, Doha, Qatar; 8 Department of Public Health, Qatar University College of Health Sciences, Doha, Qatar

**Keywords:** Autism, Intensive Care Units, Neonatal, Neonatology, Statistics, Child Health

## Abstract

**Objectives:**

Maternal diabetes is linked to adverse perinatal outcomes, but its association with neurodevelopmental delays in offspring remains unclear. This study aimed to investigate the relationship between maternal diabetes and neurodevelopmental outcomes in children born to Qatari mothers.

**Methods:**

This retrospective population-based cohort study included full-term children born in 2017 to Qatari mothers with gestational diabetes, pre-pregnancy diabetes, or no diabetes. Neurodevelopmental outcomes such as autism spectrum disorder (ASD), speech delay, and gross and fine motor delays were assessed at the age of 4 years. Data for the study were retrieved from electronic medical records.

**Results:**

Of the 5141 eligible children, 1222 (23.8%) were born to mothers with gestational diabetes mellitus (GDM), 140 (2.7%) to mothers with pre-pregnancy diabetes and 3779 (73.5%) to non-diabetic mothers. Overall, 70 (1.58%) children were diagnosed with autism, 161 (3.63%) with speech delay, 44 (0.99%) with gross motor delay and 43 (0.97%) with fine motor delay. In crude analyses, children of mothers with GDM had significantly higher odds of autism (OR 1.70, 95% CI 1.03 to 2.81) and speech delay (OR 1.44, 95% CI 1.02 to 2.04) compared with controls. However, these associations were not statistically significant after adjusting for confounders (autism: adjusted odds ratio (aOR) 1.58, 95% CI 0.94 to 2.66; speech delay: adjusted odds ratio (aOR) 1.39, 95% CI 0.97 to 2.00). No significant associations were observed between maternal diabetes and gross or fine motor delays in either crude or adjusted models.

**Conclusion:**

Despite non-significant associations, findings highlight the role of effective diabetes management and the need for region-specific, longitudinal research on child neurodevelopment.

**WHAT IS ALREADY KNOWN ON THIS TOPIC**

- The relationship between maternal diabetes mellitus and neurodevelopmental outcomes of the offspring is not clear, with some positive and negative associations in the literature.

**WHAT THIS STUDY ADDS**

- The study did not find significant associations between gestational diabetes and early childhood neurodevelopmental delays after adjusting for confounding factors.

**HOW THIS STUDY MIGHT AFFECT RESEARCH, PRACTICE OR POLICY**

- These findings highlight the importance of effective prenatal management and support and the need for future longitudinal research to explore longer-term developmental outcomes in gestational and pre-pregnancy diabetes.

## Background

 It has been widely known for years that maternal diabetes mellitus (DM), including pre-pregnancy and gestational DM, increases the risk of maternal, fetal, and neonatal mortalities and morbidities such as macrosomia, shoulder dystocia, pre-eclampsia, neonatal hypoglycaemia, hyperbilirubinaemia, congenital malformations and respiratory distress syndrome.^1 2^ Gestational diabetes mellitus (GDM) affects up to 15% of pregnant women worldwide.^3^ It is defined as glucose intolerance that first becomes recognised during pregnancy. It accounts for around 87.5% of maternal diabetes, while the rest are due to pre-pregnancy type 1 diabetes mellitus (7.5%) and pre-pregnancy type 2 diabetes mellitus (T2DM) (5%).^4 5^ It has been known that adverse short-term pregnancy and delivery outcomes are associated with diabetes mellitus.^5^

Many factors, including genetic and environmental factors, influence the structural and functional development of the brain. Environmental factors include drugs, irradiation, chemicals, infections, malnutrition, antenatal stress, maternal separation and metabolic derangements.^3 5^ Intrauterine hyperglycaemia resulting from GDM is one of these sub-optimal developmental factors. These factors can negatively impact any stage of neurodevelopment, including neurogenesis, neuronal differentiation, neuronal migration, neuronal cell apoptosis, axonal connections and synaptogenesis. These negative impacts can be through direct effects and/or indirectly through epigenetic dysregulation, and might lead to irreversible damage to the brain.^3^ GDM is associated with disturbances in insulin metabolism and insulin signalling, the inflammatory process, oxidative stress and epigenetic modification that might lead to abnormal neurodevelopment and neuropsychiatric disorders.^1 3^

There is a paucity of evidence and inconsistent results on the association between maternal pre-pregnancy and GDM and neurodevelopmental disorders in offspring.^6^,^8^ Even less evidence exists on the association between the time of exposure to maternal pre-pregnancy or GDM and the risk of neurodevelopmental disorders in the offspring.^9^,^14^ While proper management of GDM is known to improve maternal and neonatal outcomes, data on the neurodevelopment of offspring born to diabetic mothers remain scarce.^15 16^ Considering the rising prevalence of GDM, its modifiable nature, and the limited research on this topic in Qatar, this retrospective study sought to identify prognostic factors to guide therapeutic strategies and enhance epidemiological surveillance to optimise maternal glycaemic control and improve long-term outcomes.

This study aimed to investigate the long-term outcomes, including certain neurodevelopmental disorders, including autism spectrum disorder (ASD), speech delay (SD) and gross and fine motor delays in children who were born to Qatari women with GDM or pre-pregnancy diabetes.

## Methods

### Study design and data source

This retrospective study was conducted at the Women’s Wellness and Research Center (WWRC) and Rumailah Hospital (RH) at Hamad Medical Corporation (HMC) in Qatar between January 2017 and September 2021. Data for the study were retrieved from electronic medical records using the Cerner database system currently implemented across all the HMC hospitals. The dataset included maternal health information, delivery records and paediatric follow-up data.

### Study population

The study population included all term infants born between 37 weeks, 0 days and 41 weeks, 6 days of gestation in 2017 to Qatari mothers with gestational or pre-pregnancy DM only in WWRC at HMC. A total of 5195 patient records were reviewed, based on our recently published study, which assessed the maternal and neonatal outcomes in mothers with diabetes mellitus in the Qatari population.^5^ All the medical institutions in Qatar screen all pregnant women for diabetes at the first antenatal care visit. Accordingly, pregnant women are classified. That national screening is based on the 2013-WHO Criteria which states that GDM should be diagnosed at any time in pregnancy if one or more of the following criteria are met: fasting plasma glucose of 5.1 mmol/L (92 mg/dL) or greater, 1-hour plasma glucose ≥10.0 mmol/L (180 mg/dL) following a 75 g oral glucose load, or 2-hour plasma glucose 8.5–11.0 mmol/L (153–199 mg/dL) following a 75 g oral glucose load.^4 5^ Pre-pregnancy DM was defined by either type 1 or type 2 DM before the index pregnancy. Gestational age (GA) was mainly calculated using the last menstrual period (LMP), calculated from the first day of the last normal menstrual period. This assumes a 28-day cycle, with ovulation occurring on day 14. Also, an early obstetric ultrasound done in the first trimester (up to 13 6/7 weeks) was used to determine accurate GA using measurements like the crown-rump length (CRL). All the data that was used in this study were obtained from records.

Data were collected on ASD, SD and gross and fine motor delays among these children at the age of four, along with relevant maternal information. In our study, autism spectrum disorder was defined based on a formal clinical diagnosis documented in the medical record, made by a qualified developmental paediatrician, according to Diagnostic and Statistical Manual of Mental Disorders, 5th Edition (DSM-5) diagnostic criteria in place at the time of assessment. Only children with a documented ASD diagnosis were classified as having autism in the analysis. Children with isolated behavioural concerns who did not meet full diagnostic criteria for ASD were not classified as ASD cases and were instead included in the non-ASD comparison group. Thus, the ASD outcome reflects categorical clinical diagnoses rather than subclinical autistic traits or screening-level behavioural abnormalities.

Neurodevelopmental assessments for autism spectrum disorder, speech delay and motor delay were not routinely performed for all children but were initiated based on standard developmental surveillance and clinical concern. The decision to screen, refer or assess a child was not influenced by maternal gestational diabetes status. Children in both the GDM and non-GDM groups were followed through the same healthcare system, had access to the same developmental surveillance during well-child visits and were referred for assessment as needed using the same criteria. Each neurodevelopmental outcome was analysed independently, allowing children with multiple outcomes to contribute to each outcome-specific analysis.

We define neonatal hypoglycaemia based on the plasma glucose level for those who are less than 48 hours of life with plasma glucose levels <50 mg/dL (2.8 mmol/L) and for those who are greater than 48 hours of life with plasma glucose levels <60 mg/dL (3.3 mmol/L). The last HbA1C% (glycosylated haemoglobin) was checked before delivery in women with GDM and those who had pre-pregnancy DM to get an idea about glycaemic control in the preceding 3 months^5^.

Controls were all children of the same age, born in 2017 to non-diabetic Qatari mothers. No intentional selection of specific controls has been made. Exclusion criteria from the whole sample included preterm infants, multiple gestations, children with a family history of neurodevelopmental disorders, or those with congenital anomalies, chromosomal abnormalities, genetic syndromes, or neurological conditions associated with ASD and SD, such as hypoxia or toxin exposure.

### Study variables

The primary outcome was neurodevelopmental disorders, including ASD, SD and gross and fine motor delays. ASD was diagnosed in children showing warning signs during routine evaluations, using DSM-5-TR (Diagnostic and Statistical Manual of Mental Disorders, Fifth Edition, Text Revision)^17^ criteria in a multidisciplinary team led by a developmental paediatrician or psychiatrist. Speech and motor delays were identified by developmental paediatricians based on institutional and national protocols when children failed to meet age-appropriate milestones using standardised assessment tools. Speech delay was defined as expressive language abilities that are substantially and quantifiably below age expectations, resulting in functional limitations in communication, social participation, academic achievement or occupational performance. Motor delay was defined as the gross or fine motor skill abilities that are substantially and quantifiably below age expectation, resulting in functional limitation with activities of daily living, academic productivity, leisure and play.

ASD was assessed and diagnosed using the Autism Diagnostic Observation Schedule, Second Edition (ADOS-2). It is a semi-structured, standardised direct observational assessment tool that evaluates social communication, social interaction, play and restricted/repetitive behaviours consistent with ASD. Speech delay was assessed and diagnosed with the help of a speech and language therapist using the Preschool Language Scale (PLS-5). It is a standardised developmental language test assessing receptive and expressive language skills in infants and young children through structured play and interaction. Motor delay was assessed and diagnosed with the help of the physiotherapist and/or occupational therapist using assessment tools like the Bayley Scales of Infant and Toddler Development (Bayley-4) or Peabody Developmental Motor Scales (PDMS-2), which both are standardised assessment tools for motor development in young children, including reflexes, balance, grasping and visual-motor integration.

Other maternal variables collected from the electronic medical records included maternal age, GA, Body Mass Index (BMI), periconceptional and most recent glycosylated haemoglobin (HbA1c%) in pregnancy, timing of GDM diagnosis, type of GDM therapy, placental weight and other maternal pregnancy complications. We also collected neonatal data, including sex, birth weight (BW), length, and head circumference and neonatal blood sugar readings and Apgar Scores at 1, 5 and 10 min.

Data collection sheets and data analysis outputs were kept securely in a computer in the principal investigator’s (PI) office. Data were collected by research team members. It was stored as soft copies within password-locked computers at the PI office. Data were collected and de-identified, and codes were used to cover patient identifiers such as name, date of birth and health card numbers. Links to identifiers were destroyed after data collection. The de-identified study data will be stored for at least 5 years post-study completion.

### Statistical analysis

Descriptive statistics summarised baseline characteristics and the distribution of patient data. Categorical variables were summarised using frequencies and χ² tests, while continuous variables were reported as means±SD or medians (IQR), based on distribution. Univariate analysis used Yates-corrected χ² for categorical variables and analysis of variance or Kruskal-Wallis for continuous variables, based on data distribution. In multivariable analysis, logistic regression was employed to estimate crude and adjusted odds ratios (OR, adjusted odds Ratio (aOR)) along with their respective 95% CIs. Directed acyclic graphs (DAGs) were used to identify potential confounders for the multivariable model, using DAGitty Software version 3.0.11.^18^ Sensitivity analyses were conducted stratifying gestational diabetes by treatment modality as a proxy for disease severity and were restricted to participants with available treatment data. The extent of missing data was assessed for all study variables. Primary analyses were conducted using complete-case analysis for the exposure, outcomes and covariates included in the minimal adjustment set. Variables with substantial missingness were not included in the main multivariable models.

The statistical analyses were done using STATA statistical software, version 18.0, with a two-sided p value of <0.05 considered statistically significant.^19^ Additionally, the STROBE guidelines (Strengthening the Reporting of Observational Studies in Epidemiology) were used, ensuring the high-quality reporting of this observational study.^20^

## Results

### Study sample

In 2017, there were a total of 17 020 live births in WWRC. Among these, 5141 full-term neonates were born to Qatari women.^5^ Within this group, 23.8% (1222 out of 5,141) were born to mothers diagnosed with GDM, 2.7% (140 out of 5141) to mothers with pre-existing diabetes and 73.5% (3779 out of 5141) to mothers without diabetes (table 1).

**Table 1:** Demographics and baseline characteristics of the study population

Variables	Total (n=5141)
Maternal age (years)	29.65 (5.91)
Group	
GDM	1222 (23.77)
Pre-pregnancy DM	140 (2.72)
Controls	3779 (73.51)
Pre-conceptional HbA1C%	5.50 (5.20–6.10)
Time of diagnosis of GDM	
≤26 weeks gestation	348 (43.77)
>26 weeks gestation	447 (56.23)
Diabetes complications during pregnancy	
Yes	21 (1.69)
No	1224 (98.31)
Type of diabetes control	
Diet	880 (70.85)
Metformin	166 (13.37)
Insulin	81 (6.52)
Insulin and metformin	115 (9.26)
Most recent HbA1C% value during pregnancy	5.30 (5.00–5.60)
Gestational age (weeks)	39.0 (38.9–40.0)
Sex	
Male	2665 (51.84)
Female	2476 (48.16)
Neonatal hypoglycaemia	
Yes	734 (15.89)
No	3885 (84.11)
Autism	
Yes	70 (1.58)
No	4352 (98.42)
Speech delay	
Yes	161 (3.63)
No	4279 (96.37)
Gross motor developmental delay	
Yes	44 (0.99)
No	4391 (99.01)
Fine motor developmental delay	
Yes	43 (0.97)
No	4392 (99.03)
Birth weight (grams)	3091.01 (596.60)
Length (cm)	49.76 (3.30)
Head circumference (cm)	33.97 (1.91)
Apgar score at 1 min	8.93 (0.50)
Apgar score at 5 min	9.98 (0.27)
Apgar score at 10 min	10.0 (0.08)

Data are displayed as frequency and percentages n (%) for categorical measures, mean (SD) or median (IQR) for continuous measures.

DM, diabetes mellitus; GDM, gestational diabetes mellitus; HbA1C, glycosylated haemoglobin.

### DAG and confounding variables

**Figure 1:**
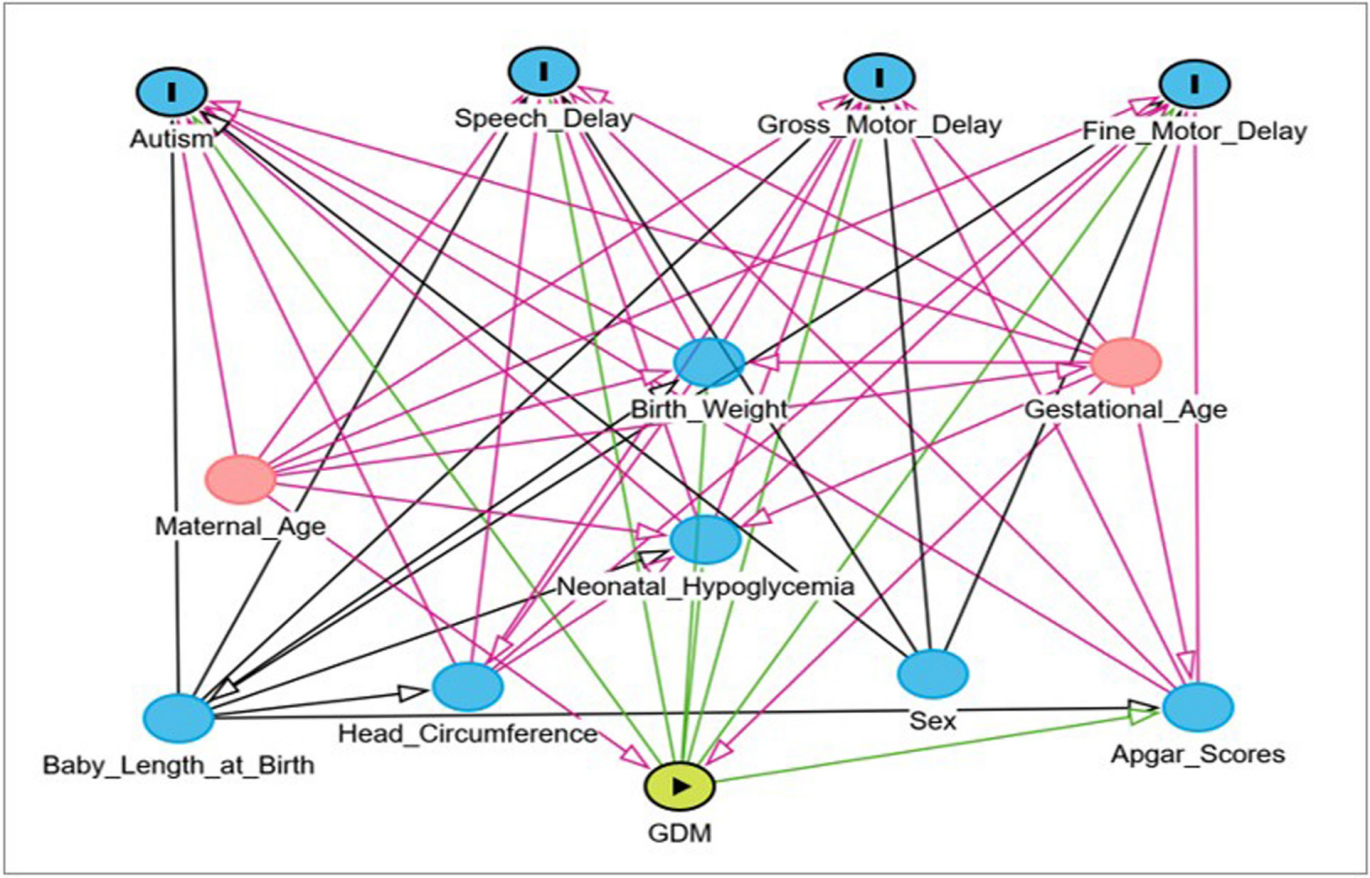
DAG of neurodevelopmental outcomes and their association with maternal diabetes. This DAG illustrates the interrelationships between potential confounders in the association between gestational diabetes and neurodevelopmental outcomes. The variables highlighted in red represent confounders included in the sufficient adjustment set in the analysis. DAG, directed acyclic graph; GDM, gestational diabetes mellitus.

Given their shared aetiology, a single DAG was developed for all neurodevelopmental outcomes to identify confounders in the association between maternal diabetes and autism, speech, gross and fine motor delays. Maternal age and GA were identified as key confounders, while child sex was treated as an effect modifier due to evidence of sex-specific susceptibility^21^ (figure 1; online supplemental tables 4–7).

### Sensitivity analysis by gestational diabetes severity

In sensitivity analyses stratified by gestational diabetes severity, no statistically significant differences were observed between diet-controlled and medication-treated GDM across neurodevelopmental outcomes. Effect estimates were similar in magnitude and direction, with no evidence of increasing risk with greater GDM severity.

### Demographic characteristics of the study population

**Figure 2:**
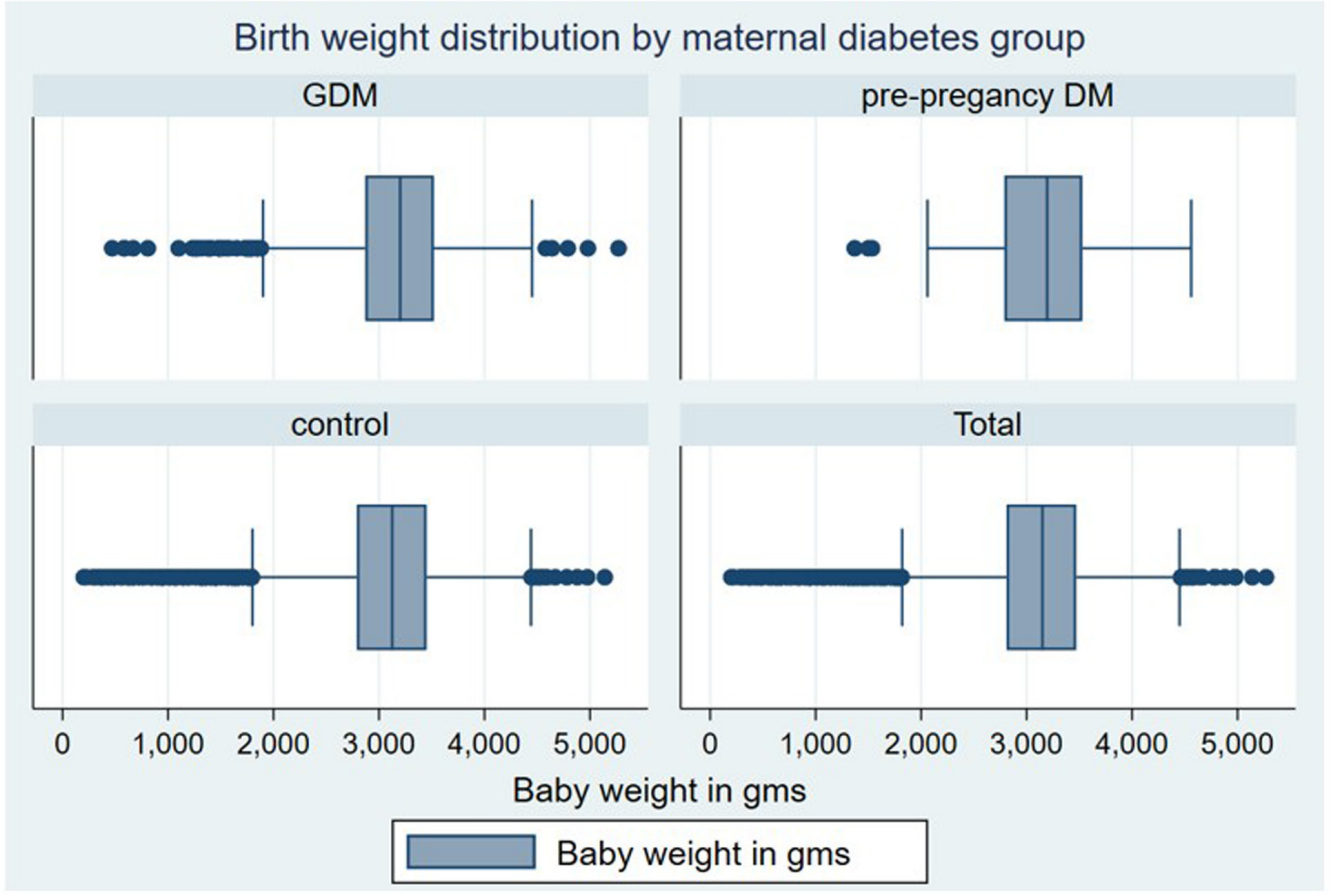
Distribution of neonatal birth weight by maternal diabetes group. This figure presents the distribution of neonatal birth weight across three maternal diabetes categories: control (no diabetes), gestational diabetes mellitus (GDM) and pre-pregnancy diabetes. The box plots display each group’s median, IQR and potential outliers. DM, diabetes mellitus.

Table 1 summarises the maternal and neonatal birth characteristics of the study population. The mean birth weight of the newborns was 3091 grams (figure 2), with a 1:1 male-to-female ratio. Most children were born at a median GA of 39 weeks, and the mean APGAR scores were 8.9 at 1 min, 9.9 at 5 min and 10 at 10 min. The mothers had an average age of around 30 years. Among them, 43.8% were diagnosed with GDM at or before 26 weeks of gestation, while 56.2% received the diagnosis after 26 weeks. In the majority of cases, elevated glucose levels were managed mainly through dietary interventions (~70%) or with metformin (13.3%).

**Figure 3:**
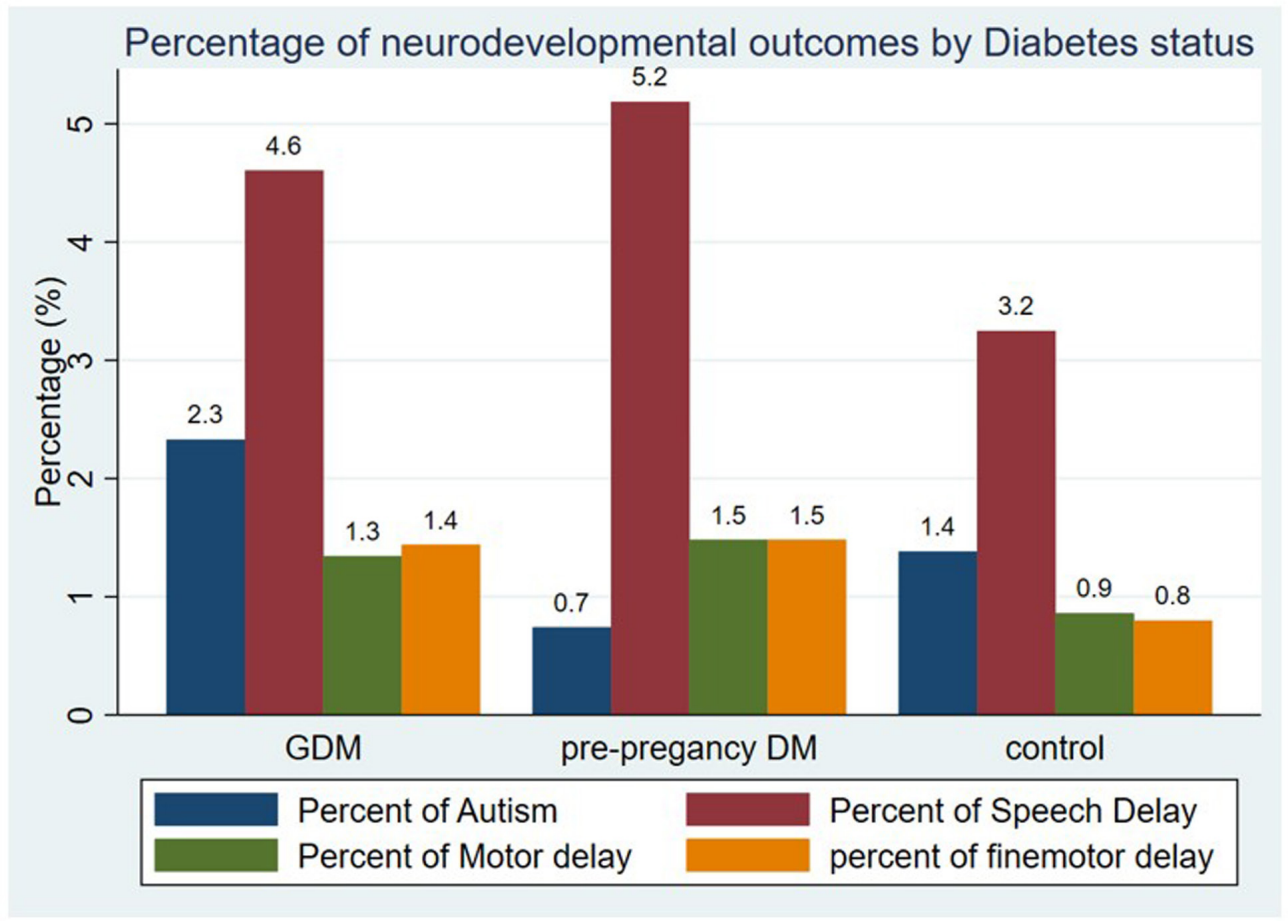
Neurodevelopmental outcomes in children by maternal diabetes status. This bar chart displays the percentage of children diagnosed with autism, speech delay, gross motor delay and fine motor delay across three maternal diabetes groups: control (no diabetes), gestational diabetes mellitus (GDM) and pre-pregnancy diabetes. DM, diabetes mellitus.

Table 2 summarises differences across diabetes groups. Mothers with GDM had lower HbA1c levels (5.3% preconception, 5.2% during pregnancy) than those with pre-pregnancy diabetes (7.0% and 6.0%). GDM was mainly managed with diet (79.8%) or metformin (13.7%), while pre-pregnancy diabetes was treated with insulin alone (23.6%) or with metformin (65%). Pregnancy complications were more common in pre-pregnancy diabetes (3%) than in GDM (1.5%). Neonatal hypoglycaemia was highest in pre-pregnancy diabetes (64.3%), followed by GDM (41.3%) and controls (5.7%). Autism was more frequent in the GDM group (2.33%) than in pre-pregnancy diabetes (0.7%) or controls (1.4%). Speech and gross motor delays were most common in pre-pregnancy diabetes (5.2%, 1.5%), followed by GDM (4.6%, 1.3%) and controls (3.2%, 0.9%). Fine motor delay was similar in both diabetes groups (1.4%) and higher than in controls (0.8%) (figure 3).

**Table 2:** Demographics and baseline characteristics of the study population by diabetes group

	GDM	Pre-pregnancy DM	Control	Total
	n=1222	n=140	n=3779	n=5141
HbA1C during pregnancy	5.20 (5.00–5.50)	6.00 (5.50–6.60)		5.30 (5.00–5.60)
Pre-conceptional HbA1C	5.30 (5.10–5.50)	7.00 (6.100–8.600)		5.500 (5.200–6.100)
Complications in pregnancy				
No	1088 (98.46)	136 (97.14)		1224 (98.31)
Yes	17 (1.54)	4 (2.86)		21 (1.69)
Type of therapy				
Diet	879 (79.76)	1 (0.71)		880 (70.85)
Metformin	151 (13.70)	15 (10.71)		166 (13.37)
Insulin	48 (4.36)	33 (23.57)		81 (6.52)
Metformin & insulin	24 (2.18)	91 (65.00)		115 (9.26)
Gestational age (weeks)	39 (38–40)	38 (37–38)	39 (38–40)	39 (38–40)
Maternal age (years)	31.62 (5.88)	34.81 (6.10)	28.82 (5.67)	29.65 (5.91)
Sex				
Male	640 (52.37)	59 (42.14)	1966 (52.02)	2665 (51.84)
Female	582 (47.63)	81 (57.86)	1813 (47.98)	2476 (48.16)
Birth weight (gm)	3162.67 (571.90)	3131.29 (568.82)	3066.34 (603.58)	3091.01 (596.60)
Length of baby at birth (cm)	49.88 (3.39)	49.51 (2.70)	49.73 (3.29)	49.76 (3.30)
Birth head circumference (cm)	34.06 (2.27)	34.10 (1.71)	33.49 (4.26)	33.64 (3.84)
Neonatal hypoglycaemia				
No	640 (58.72)	50 (35.71)	3195 (94.28)	3885 (84.11)
Yes	450 (41.28)	90 (64.29)	194 (5.72)	734 (15.89)
APGAR score at 1 min	8.93 (0.51)	8.78 (0.83)	8.94 (0.48)	8.93 (0.50)
APGAR score at 5 min	9.97 (0.32)	9.86 (0.47)	9.98 (0.25)	9.98 (0.27)
APGAR score at 10 min	9.99 (0.04)	10.0 (0.0)	9.99 (0.09)	9.99 (0.08)
Autism				
No	1007 (97.67)	134 (99.26)	3211 (98.62)	4352 (98.42)
Yes	24 (2.33)	1 (0.74)	45 (1.38)	70 (1.58)
Speech delay				
No	994 (95.39)	128 (94.82)	3157 (96.75)	4279 (96.37)
Yes	48 (4.61)	7 (5.19)	106 (3.25)	161 (3.63)
Gross motor delay				
No	1028 (98.66)	133 (98.52)	3230 (99.14)	4391 (99.01)
Yes	14 (1.34)	2 (1.48)	28 (0.86)	44 (0.99)
Fine motor delay				
No	1027 (98.56)	133 (98.52)	3232 (99.20)	4392 (99.03)
Yes	15 (1.44)	2 (1.48)	26 (0.80)	43 (0.97)

Data are displayed as frequency and percentages n (%) for categorical measures, mean (SD) or median (IQR) for continuous measures.

DM, diabetes mellitus; GDM, gestational diabetes mellitus; HbA1C, glycosylated haemoglobin.

### Association between maternal diabetes and autism

In the study population, the prevalence of autism was 1.58% (95% CI 1.25 to 2.0; table 1). Crude analysis showed that children born to mothers with GDM had 70% higher odds of developing autism compared with the control group, and this association was statistically significant (OR 1.70, 95% CI 1.03 to 2.81; table 3). However, after adjusting for potential confounders in the multivariable model (maternal age, GA and sex), the association was no longer statistically significant (aOR 1.58, 95% CI 0.94 to 2.66; table 7).

**Table 3:** Maternal and neonatal characteristics of the study population by autism (univariate analysis)

Autism					
	Yes	No	Total	Univariate model	P value
	n=70	n=4352	n=4422	OR (95% CI)	
Diabetes in pregnancy					
GDM	24 (34.29)	1007 (23.14)	1031 (23.31)	1.70 (1.03 to 2.81)	0.038
Pre-pregnancy DM	1 (1.43)	134 (3.08)	135 (3.05)	0.53 (0.07 to 3.89)	0.54
Control	45 (64.29)	3211 (73.78)	3256 (73.63)	1.00	
Pregnancy HbA1C value	5.20 (5.10–5.50)	5.30 (5.00–5.60)	5.30 (5.00–5.60)	0.72 (0.32 to 1.61)	0.42
Gestational age (weeks)	39 (38–40)	39 (38–40)	39 (38–40)	0.92 (0.77 to 1.11)	0.40
Maternal age (years)	30.46 (5.87)	29.56 (5.91)	29.57 (5.91)	1.03 (0.99 to 1.07)	0.21
Pre-conceptional HbA1C	5.35 (5.20–5.50)	5.50 (5.20–6.10)	5.50 (5.20–6.10)	0.42 (0.10 to 1.85)	0.254
Time of diagnosis of GDM					
<26 weeks	7 (35.0)	315 (43.63)	322 (43.40)	1.44 (0.57 to 3.64)	0.45
>26 weeks	13 (65.0)	407 (56.37)	420 (56.60)	1.00	
Type of therapy					
Diet	19 (76.0)	802 (70.66)	821 (70.78)	1.00	
Metformin	4 (16.0)	148 (13.04)	152 (13.10)	1.14 (0.38 to 3.40)	0.81
Insulin	1 (4.0)	76 (6.69)	77 (6.64)	0.56 (0.07 to 4.21)	0.57
Metformin & insulin	1 (4.0)	109 (9.60)	110 (9.48)	0.39 (0.05 to 2.93)	0.36
Sex					
Male	49 (70.0)	2218 (50.97)	2267 (51.27)	2.24 (1.34 to 3.76)	0.002
Female	21 (30.0)	2134 (49.04)	2155 (48.73)		
Birth weight (gm)	3243.07 (448.30)	3202.32 (448.91)	3202.97 (448.88)	1.00 (0.99 to 1.00)	0.45
Length of baby at birth (cm)	50.08 (2.02)	50.28 (2.59)	50.27 (2.58)	0.98 (0.91 to 1.05)	0.52
Head circumference at birth (cm)	34.42 (1.40)	34.26 (1.56)	34.26 (1.56)	1.08 (0.92 to 1.26)	0.36
Neonatal hypoglycaemia					
No	57 (82.61)	3662 (84.61)	3719 (84.58)		
Yes	12 (17.39)	666 (15.39)	678 (15.42)	1.16 (0.62 to 2.17)	0.65
APGAR score at 1 min	8.99 (0.12)	8.94 (0.48)	8.94 (0.48)	1.68 (0.48 to 5.94)	0.42
APGAR score at 5 min	10.0 (0.0)	9.98 (0.24)	9.98 (0.24)	1.00	–
APGAR score at 10 min	10.0 (0.0)	9.99 (0.08)	9.99 (0.08)	1.00	–

Data are displayed as frequency and percentages n (%) for categorical measures, mean (SD) or median (IQR) for continuous measures, missing data as percentages n (%). P values are calculated excluding the missing.

DM, diabetes mellitus; GDM, gestational diabetes mellitus; HbA1C, glycosylated haemoglobin.

For the group with pre-pregnancy diabetes, no statistically significant association with autism was observed in either the crude or adjusted models (crude: OR 0.53, 95% CI 0.53 to 3.89; table 3; adjusted: aOR 0.46, 95% CI 0.06 to 3.52; table 7). Additionally, no interactions were observed between maternal diabetes and maternal age, GA or child sex.

In crude analyses, higher odds of autism were observed among male children (OR 2.24, 95% CI 1.34 to 3.76; table 3); however, these estimates were not interpreted causally.

### Association between maternal diabetes and speech delay

The prevalence of speech delay among children in the study population was 3.63% (95% CI 3.11 to 4.22; table 1). In the crude analysis, children born to mothers with gestational diabetes had 44% higher odds of speech delay compared with the control group (OR 1.44, 95% CI 1.02 to 2.04; table 4). After adjusting for potential confounders (maternal age, GA and sex), the odds remained elevated but were no longer statistically significant (aOR 1.39, 95% CI 0.97 to 2.0; table 7).

**Table 4:** Maternal and neonatal characteristics of the study population by speech delay (univariate analysis)

Speech delay					
	Yes	No	Total	Univariate model	P value
	n=161	n=4279	n=4440	OR (95% CI)	
Diabetes in pregnancy					
GDM	48 (29.81)	994 (23.23)	1042 (23.47)	1.44 (1.02 to 2.04)	0.04
Pre-pregnancy DM	7 (4.35)	128 (2.99)	135 (3.04)	1.63 (0.74 to 3.57)	0.22
Control	106 (65.84)	3157 (73.78)	3263 (73.49)	1.00	
Pregnancy HbA1C value	5.20 (5.00–5.60)	5.30 (5.00–5.60)	5.30 (5.00–5.60)	0.92 (0.57 to 1.47)	0.73
Gestational age (weeks)	39 (38–40)	39 (38–40)	39 (38–40)	0.87 (0.77 to 0.99)	0.03
Maternal age (years)	29.832 (6.365)	29.572 (5.891)	29.582 (5.908)	1.01 (0.98 to 1.03)	0.58
Pre-conceptional HbA1C	5.60 (5.35–5.80)	5.500 (5.20–6.10)	5.50 (5.20–6.10)	0.90 (0.64 to 1.27)	0.56
Time of diagnosis of GDM					
<26 weeks	15 (37.50)	313 (44.09)	328 (43.73)	1.31 (0.68 to 2.53)	0.42
>26 weeks	25 (62.50)	397 (55.92)	422 (56.27)	1.00	
Complications during pregnancy					
No	53 (98.15)	1101 (98.30)	1154 (98.30)	1.00	
Yes	1 (1.85)	19 (1.70)	20 (1.70)	1.09 (0.14 to 8.32)	0.93
Type of therapy					
Diet	36 (66.67)	792 (70.90)	828 (70.71)	1.00	
Metformin	8 (14.82)	146 (13.07)	154 (13.15)	1.20 (0.55 to 2.65)	0.64
Insulin	3 (5.56)	75 (6.71)	78 (6.66)	0.88 (0.26 to 2.93)	0.84
Metformin & insulin	7 (12.96)	104 (9.31)	111 (9.48)	1.48 (0.64 to 3.41)	0.36
Sex					
Male	110 (68.32)	2170 (50.71)	2280 (51.35)	2.09 (1.49 to 2.94)	<0.001
Female	51 (31.68)	2109 (49.29)	2160 (48.65)		
Birth weight (gm)	3182.45 (489.67)	3203.52 (447.42)	3202.76 (448.98)	0.99 (0.99 to 1.00)	0.56
Length of baby at birth (cm)	50.06 (2.66)	50.28 (2.58)	50.27 (2.58)	0.97 (0.92 to 1.02)	0.28
Head circumference at birth (cm)	34.15 (1.56)	34.26 (1.56)	34.26 (1.56)	0.96 (0.88 to 1.05)	0.37
Neonatal hypoglycaemia					
No	127 (79.38)	3605 (84.72)	3732 (84.53)	1.00	
Yes	33 (20.62)	650 (15.28)	683 (15.47)	1.44 (0.97 to 2.13)	0.07
APGAR score at 1 min	8.83 (0.94)	8.94 (0.48)	8.93 (0.51)	0.79 (0.65 to 0.95)	0.015
APGAR score at 5 min	9.90 (0.75)	9.98 (0.24)	9.98 (0.28)	0.70 (0.54 to 0.91)	0.008
APGAR score at 10 min	9.98 (0.18)	9.99 (0.080)	9.99 (0.09)	0.51 (0.24 to 1.09)	0.08

Data are displayed as frequency and percentages n (%) for categorical measures, mean (SD) or median (IQR) for continuous measures, and missing data as percentages n (%). P values are calculated excluding the missing.

DM, diabetes mellitus; GDM, gestational diabetes mellitus; HbA1C, glycosylated haemoglobin.

Children born to mothers with pre-pregnancy diabetes also showed higher odds of speech delay compared with controls; however, the association was not statistically significant in either the crude (OR 1.63, 95% CI 0.74 to 3.57; table 4) or adjusted models (aOR 1.45, 95% CI 0.62 to 3.39; table 7). No interactions were observed between maternal diabetes and maternal age, GA or child sex.

Several other factors in the crude analysis showed unadjusted associations with speech delay such as GA, APGAR scores at 1 and 5 min and infant gender (GA: OR 0.87, 95% CI 0.77 to 0.99; APGAR at 1 min: OR 0.79, 95% CI 0.65 to 0.95; APGAR at 5 min: OR 0.70, 95% CI 0.54 to 0.91; gender: 2.09, 95% CI 1.49 to 2.94; table 4). However, since they are not our primary exposure, these estimates were not interpreted causally.

### Association between maternal diabetes and gross motor delay

The prevalence of gross motor delay was 0.99% (95% CI 0.15 to 0.74; table 1). No significant association was found between GDM and gross motor delay in either crude (OR 1.57, 95% CI 0.82 to 2.99; table 5) or adjusted analyses (aOR 1.33, 95% CI 0.68 to 2.59; table 7). Similarly, pre-pregnancy diabetes showed no significant association (crude: OR 1.73, 95% CI 0.41 to 7.36; adjusted: aOR 1.20, 95% CI 0.26 to 5.63). No interactions were observed between maternal diabetes and maternal age, GA or child sex.

**Table 5:** Maternal and neonatal characteristics of the study population by gross motor delay (univariate analysis)

Gross motor delay					
	Yes	No	Total	Univariate model	P value
	n=44	n=4391	n=4435	OR (95% CI)	
Diabetes in pregnancy					
GDM	14 (31.82)	1028 (23.41)	1042 (23.50)	1.57 (0.82 to 2.99)	0.17
Pre-pregnancy DM	2 (4.55)	133 (3.03)	135 (3.04)	1.73 (0.41 to 7.36)	0.46
Control	28 (63.64)	3230 (73.56)	3258 (73.46)	1.00	
Pregnancy HbA1C value	5.20 (5.10–5.90)	5.30 (5.00–5.60)	5.30 (5.00–5.60)	1.20 (0.63 to 2.28)	0.58
Gestational age (weeks)	39 (38–40)	39 (38–40)	39 (38–40)	0.92 (0.73 to 1.16)	0.49
Maternal age (years)	31.75 (6.75)	29.57 (5.89)	29.59 (5.91)	1.06 (1.01 to 1.12)	0.015
Pre-conceptional HbA1C	5.45 (5.30–5.60)	5.50 (5.20–6.10)	5.50 (5.20–6.10)	0.88 (0.53 to 1.46)	0.61
Time of diagnosis of GDM					
<26 weeks	5 (55.56)	323 (43.53)	328 (43.68)	1.62 (0.43 to 6.09)	0.47
>26 weeks	4 (44.44)	419 (56.47)	423 (56.33)	1.00	1.00
Complications during pregnancy					
No	14 (93.33)	1140 (98.36)	1154 (98.30)	1.00	1.00
Yes	1 (6.67)	19 (1.64)	20 (1.70)	4.29 (0.54 to 34.26)	0.17
Type of therapy					
Diet	9 (60.0)	819 (70.85)	828 (70.71)	1.00	1.00
Metformin	1 (6.67)	153 (13.24)	154 (13.15)	0.59 (0.07 to 4.73)	0.62
Insulin	2 (13.33)	76 (6.57)	78 (6.66)	2.39 (0.51 to 11.28)	0.27
Metformin & insulin	3 (20.00)	108 (9.34)	111 (9.48)	2.53 (0.67 to 9.48)	0.17
Sex					
Male	24 (54.55)	2254 (51.33)	2278 (51.36)	1.14 (0.63 to 2.07)	0.67
Female	20 (45.46)	2137 (48.67)	2157 (48.64)	1.00	
Birth weight (gm)	3077.39 (511.88)	3204.31 (448.15)	3203.05 (448.94)	0.99 (0.99 to 1.00)	0.06
Length of baby at birth (cm)	49.16 (3.03)	50.29 (2.58)	50.27 (2.58)	0.93 (0.89 to 0.98)	0.006
Head circumference at birth (cm)	33.84 (1.63)	34.27 (1.56)	34.26 (1.56)	0.91 (0.82 to 1.01)	0.07
Neonatal hypoglycaemia					
No	37 (84.09)	3691 (84.54)	3728 (84.54)	1.00	
Yes	7 (15.91)	675 (15.46)	682 (15.47)	1.03 (0.46 to 2.33)	0.94
APGAR score at 1 min	8.2 (1.83)	8.94 (0.47)	8.93 (0.51)	0.56 (0.47 to 0.67)	<0.001
APGAR score at 5 min	9.58 (61.48)	9.98 (0.23)	9.98 (0.28)	0.53 (0.39 to 0.74)	<0.001
APGAR score at 10 min	9.93 (0.35)	9.99 (0.08)	9.99 (0.09)	0.37 (0.17 to 0.81)	0.013

Data are displayed as frequency and percentages n (%) for categorical measures, mean (SD) or median (IQR) for continuous measures, and missing data as percentages n (%). P values are calculated excluding the missing.

DM, diabetes mellitus; GDM, gestational diabetes mellitus; HbA1C, glycosylated haemoglobin.

On the other hand, several neonatal and maternal characteristics were associated with gross motor delay. In the crude analysis, longer birth length was associated with a 7% reduction in the odds of gross motor delay (OR 0.93, 95% CI 0.89 to 0.98; table 5). Additionally, higher APGAR scores at 1, 5 and 10 min were linked to significantly lower odds of gross motor delay by 44%, 47% and 63%, respectively (APGAR at 1 min: OR 0.56, 95% CI 0.47 to 0.67; APGAR at 5 min: OR 0.53, 95% CI 0.39 to 0.74; APGAR at 10 min: OR 0.37, 95% CI 0.17 to 0.81; table 5).

Maternal age was also a significant factor. Older maternal age was associated with increased odds of gross motor delay, with a 6% rise in risk in both crude and adjusted models (crude & adjusted: OR 1.06, 95% CI 1.01 to 1.12; table 5, online supplemental table 6).

On the other hand, several maternal and neonatal characteristics showed unadjusted associations with gross motor delay (table 5). Longer birth length and higher Apgar scores at 1, 5 and 10 min were associated with lower odds of gross motor delay (OR 0.93, 95% CI 0.89 to 0.98; APGAR at 1 min: OR 0.56, 95% CI 0.47 to 0.67; APGAR at 5 min: OR 0.53, 95% CI 0.39 to 0.74; APGAR at 10 min: OR 0.37, 95% CI 0.17 to 0.81; table 5). A higher maternal age was associated with higher unadjusted odds (OR 1.06, 95% CI 1.01 to 1.12; table 5). No causal inference was based on these associations.

### Association between maternal diabetes and fine motor delay

The proportion of children diagnosed with fine motor delay in the study population was 0.97% (95% CI 0.72 to 1.30; table 1). Maternal gestational diabetes was associated with increased odds of fine motor delay, and the result was close to statistical significance in the crude analysis (OR 1.82, 95% CI 0.96 to 3.44; table 6). However, after adjusting for potential confounders (maternal age, GA and sex), this association was no longer observed (aOR 1.44, 95% CI 0.74 to 2.79; table 7). A similar pattern was noted among mothers with pre-pregnancy diabetes, where no significant association was found in either the crude (OR 1.87, 95% CI 0.44 to 7.96; table 6) or adjusted models (aOR 1.01, 95% CI 0.21 to 4.77; table 7). Additionally, no interactions were observed between maternal diabetes and maternal age, GA or child sex.

**Table 6:** Maternal and neonatal characteristics of the study population by fine motor delay (univariate analysis)

Fine motor delay					
	Yes	No	Total	Univariate model	P value
	n=43	n=4392	n=4435	OR (95% CI)	
Diabetes in pregnancy					
GDM	15 (34.88)	1027 (23.38)	1042 (23.50)	1.82 (0.96 to 3.44)	0.07
Pre-pregnancy DM	2 (4.65)	133 (3.028)	135 (3.04)	1.87 (0.44 to 7.96)	0.40
Control	26 (60.47)	3232 (73.59)	3258 (73.46)	1.00	
Pregnancy HbA1C value	5.30 (5.20–5.60)	5.30 (5.00–5.60)	5.30 (5.00–5.60)	1.15 (0.60 to 2.20)	0.68
Gestational age	39 (38–40)	39 (38–40)	39 (38–40)	0.82 (0.65 to 1.04)	0.10
Maternal age	32.16 (6.80)	29.56 (5.89)	29.59 (5.91)	1.07 (1.02 to 1.13)	0.01
Pre-conceptional HbA1C	5.40 (5.30–5.60)	5.50 (5.20–6.10)	5.50 (5.20–6.10)	0.87 (0.51 to 1.46)	0.59
Time of diagnosis of GDM					
<26 weeks	7 (70.00)	321 (43.32)	328 (43.68)	3.05 (0.78 to 11.90)	0.11
>26 weeks	3 (30.00)	420 (56.68)	423 (56.33)	1.00	1.00
Complications during pregnancy					
No	15 (93.75)	1139 (98.36)	1154 (98.30)	1.00	1.00
Yes	1 (6.25)	19 (1.64)	20 (1.70)	3.99 (0.50 to 31.81)	0.19
Type of therapy					
Diet	10 (62.50)	818 (70.82)	828 (70.71)	1.00	1.00
Metformin	2 (12.50)	152 (13.16)	154 (13.15)	1.08 (0.23 to 4.96)	0.93
Insulin	1 (6.250%)	77 (6.667%)	78 (6.661%)	1.06 (0.13 to 8.41)	0.95
Metformin & insulin	3 (18.75)	108 (9.35)	111 (9.48)	2.27 (0.62 to 8.39)	0.22
Sex					
Male	25 (58.14)	2254 (51.32)	2279 (51.39)	1.32 (0.72 to 2.42)	0.38
Female	18 (41.86)	2138 (48.68)	2156 (48.61)	1.00	1.00
Birth weight (gm)	3089.65 (499.62)	3203.97 (448.47)	3202.86 (449.07)	0.99 (0.99 to 1.00)	0.09
Length of baby at birth (cm)	49.40 (3.25)	50.28 (2.57)	50.27 (2.58)	0.94 (0.89 to 0.99)	0.03
Head circumference at birth (cm)	33.93 (1.73)	34.26 (1.56)	34.26 (1.56)	0.92 (0.82 to 1.03)	0.15
Neonatal hypoglycaemia					
No	36 (83.72)	3691 (84.52)	3727 (84.51)	1.00	1.00
Yes	7 (16.28)	676 (15.48)	683 (15.49)	1.06 (0.47 to 2.40)	0.89
APGAR score at 1 min	8.31 (1.81)	8.94 (0.47)	8.93 (0.51)	0.57 (0.48 to 0.69)	<0.01
APGAR score at 5 min	9.59 (1.50)	9.98 (0.23)	9.98 (0.28)	0.55 (0.40 to 0.74)	<0.01
APGAR score at 10 min	9.95 (0.32)	9.99 (0.08)	9.99 (0.09)	0.43 (0.20 to 0.97)	0.04

Data are displayed as frequency and percentages n (%) for categorical measures, mean (SD) or median (IQR) for continuous measures, and missing data as percentages n (%). P values are calculated excluding the missing.

DM, diabetes mellitus; GDM, gestational diabetes mellitus; HbA1C, glycosylated haemoglobin.

**Table 7:** Maternal and neonatal characteristics of the study population by developmental outcomes (univariate and multivariate analyses)

Gestational group	OR (95% CI)	P value
**Autism**		
GDM		
Crude	1.70 (1.03 to 2.81)	0.038
Adjusted	1.58 (0.94 to 2.66)	0.08
Pre-pregnancy diabetes		
Crude	0.53 (0.07 to 3.89)	0.54
Adjusted	0.46 (0.06 to 3.52)	0.45
**Speech delay**		
GDM		
Crude	1.44 (1.02 to 2.04)	0.04
Adjusted	1.39 (0.97 to 2.00)	0.07
Pre-pregnancy diabetes		
Crude	1.63 (0.74 to 3.57)	0.22
Adjusted	1.45 (0.62 to 3.39)	0.39
**Gross motor delay**		
GDM		
Crude	1.57 (0.82 to 2.99)	0.17
Adjusted	1.33 (0.68 to 2.59)	0.40
Pre-pregnancy diabetes		
Crude	1.73 (0.41 to 7.36)	0.46
Adjusted	1.20 (0.26 to 5.63)	0.82
**Fine motor delay**		
GDM		
Crude	1.82 (0.96 to 3.44)	0.07
Adjusted	1.44 (0.74 to 2.79)	0.28
Pre-pregnancy diabetes		
Crude	1.87 (0.44 to 7.96)	0.4
Adjusted	1.01 (0.21 to 4.77)	0.99

Crude, univariate logistic regression analysis; adjusted, multivariable logistic regression analysis; all four outcome variables were adjusted for maternal age, gestational age and sex.

DM, diabetes mellitus; GDM, gestational diabetes mellitus; HbA1C, glycosylated haemoglobin.

Crude analyses suggested unadjusted associations between selected neonatal and maternal characteristics and fine motor delay such as birth length (OR 0.94, 95% CI 0.89 to 0.99; table 6), APGAR scores at 1, 5 and 10 min (APGAR at 1 min: OR 0.57, 95% CI 0.48 to 0.69; APGAR at 5 min: OR 0.55, 95% CI 0.40 to 0.74; APGAR at 10 min: OR 0.43, 95% CI 0.20 to 0.97; table 6) and maternal age (crude: OR 1.07, 95% CI 1.02 to 1.13; table 6). These results are presented descriptively, with inference based on adjusted models in which gestational diabetes was the primary exposure.

## Discussion

This study examined the association between maternal diabetes and neurodevelopmental outcomes in full-term children born to Qatari mothers. Although the prevalence of autism, speech delay and motor delays was higher among children of mothers with gestational or pregestational diabetes compared with those born to non-diabetic mothers, these associations did not reach statistical significance after adjusting for potential confounders.

### Prevalence of maternal diabetes and neurodevelopmental outcomes

Examining the prevalence rates of maternal diabetes and neurodevelopmental outcomes within the study cohort is essential for interpreting the observed findings. In our study, the prevalence of gestational diabetes was 23.8%, which is higher than the previously recorded rate in Qatar (20.7%).^22^ The prevalence of gestational diabetes in the Middle East and North Africa (MENA) region was 13%, and in the Gulf Cooperation Council (GCC), it was 27.5%,^22^ and the global rate was 14%.^23^ The prevalence of pre-pregnancy diabetes in our study was 2.7%, which exceeds the MENA region’s rate of 2.4%, and global rates for pre-existing type 1 and type 2 diabetes (0.3% and 0.2%, respectively).^24^ These elevated rates of pre-pregnancy diabetes can be due to genetic predispositions, lifestyle factors, better healthcare access and cultural practices. Addressing these factors through further public health initiatives, quality improvement projects and large research studies is crucial to mitigating the rising incidence of diabetes in Qatar.

The burden of autism in our study was 1.6%, which is higher than the 1.14% previously documented in a 2019 study conducted in Qatar.^25^ Although these rates are lower than those reported in Saudi Arabia (2.5%), they are higher than the pooled rates in the MENA region (0.13%)^26^ and globally (0.77%).^27^ These higher rates can largely be attributed to better awareness, more accessible and advanced healthcare services, and higher diagnostic rates. The prevalence of speech delay in our study (3.6%) was much lower than rates reported in neighbouring countries, such as Saudi Arabia (24.5%), Iran (6.5%)^28^ and Egypt (5.3%).^29^ Similarly, the prevalence of motor delay (~1%) in our cohort was lower than rates reported in Egypt (1.5%).^29^ Given the limited data on the prevalence of speech and motor delays in the region, additional studies are needed to further understand these outcomes and their contributing factors.

### Maternal diabetes exposure and risk of neurodevelopmental outcomes

Our findings partially align with previous literature suggesting an elevated risk of ASD in the offspring of mothers with diabetes. Several systematic reviews have reported a higher risk of ASD among children born to mothers with GDM compared with those with pre-diabetes or without diabetes.^6 30^ In our study, GDM was associated with an increased risk of autism; however, this association was no longer significant after adjustment for maternal age, GA and child sex. This attenuation may be explained by the fact that advanced maternal age,^31^ male sex^32^ and preterm birth^33^ are proposed risk factors for ASD and are also more prevalent among pregnancies complicated by GDM, suggesting that the observed unadjusted association may be partially attributable to these shared risk factors. This is consistent with a 2022 systematic review in which the significant unadjusted association became non-significant after adjustment.^31^ Another review also reported a link between GDM and ASD but noted high heterogeneity and risk of bias among the included studies.^32^ Given the mixed and primarily observational nature of the existing evidence, prospective studies are needed to clarify the association between maternal diabetes and ASD.

The association between maternal diabetes and neurodevelopmental outcomes such as speech and motor delays has been less studied compared with conditions like ASD and attention-deficit/hyperactivity disorder (ADHD), resulting in limited evidence to draw definitive conclusions. Available studies suggest a potential increased risk of speech delay among the offspring of women with gestational or pregestational diabetes.^33^ In our study, gestational diabetes was significantly associated with speech delay in unadjusted models; however, this association did not persist after adjustment for potential confounders. This finding is consistent with previous research, where the observed associations were attenuated after controlling for factors such as fetal sex, ethnicity, socioeconomic status and maternal health characteristics.^34^

Regarding motor delay, existing literature indicates an elevated risk of both fine and gross motor delays in children of mothers with gestational and pre-existing diabetes.^33 35^ However, our study found no statistically significant association between gestational diabetes and motor delay. These findings agree with results from a recent systematic review,^21^ which noted that associations between maternal diabetes and developmental delays were often confounded or masked by coexisting maternal conditions such as gestational hypertension and elevated pre-pregnancy BMI.

The absence of statistically significant associations between gestational diabetes and neurodevelopmental outcomes may be partly attributable to the predominantly mild GDM profile of the cohort, as sensitivity analyses stratified by disease severity demonstrated no associations across diet-controlled and medication-treated GDM. In addition, most cases were diagnosed after 26 weeks, managed through dietary interventions, and had well-controlled HbA1c levels, conditions unlikely to trigger the inflammatory pathways linked to neurodevelopmental disruption.^36^ In addition, Qatar’s comprehensive antenatal and postnatal care may have further moderated the risks. Unlike many previous studies relying on parent-reported questionnaires, our use of standardised clinical assessments likely produced more accurate and conservative estimates, contributing to differences in findings.

Additionally, the associations in our study were likely influenced by key factors. Increased maternal age was significantly associated with motor delay in our study, aligning with findings that advanced maternal age (≥40 years) is a strong predictor of neurodevelopmental impairment, possibly through mechanisms involving reduced oocyte quality, placental dysfunction and higher prevalence of gestational complications.^37^ Higher Apgar scores, particularly at 5 min, are indicative of adequate neonatal adaptation, effective oxygenation, and absence of birth-related hypoxia or asphyxia. The Apgar score is a reflection of the well-being of the newborn. In itself, it is not an exposure. Studies have shown that higher Apgar scores reflect favourable intrauterine and perinatal conditions, which are associated with a reduced need for intensive interventions and healthier neurodevelopmental outcomes.^38 39^ Moreover, neurodevelopmental impairments differed by child sex, with a higher prevalence observed among males, consistent with current literature documenting sex-based susceptibility to autism and language-related delays.^37^ In our analyses, child sex was not treated as a confounder but was included as an effect modifier.

### Strengths of the study

This study has several strengths. It is the first in Qatar and the Gulf region to concurrently examine gestational diabetes and multiple neurodevelopmental outcomes (autism, speech and motor delays), offering a comprehensive assessment of early childhood development. The population-based design, focused on Qatari nationals in a high-prevalence setting for GDM, enhances both internal validity and regional relevance. In addition, the use of standardised clinical assessments, rather than parent-reported questionnaires, minimised reporting bias and improved diagnostic accuracy. Furthermore, the application of DAGs enabled a theory-driven, evidence-based approach to confounder selection, strengthening the robustness of the adjusted models.

### Limitations of the study

This study has several limitations. First, its cross-sectional design limits the ability to infer causality between gestational diabetes and neurodevelopmental outcomes. Second, the sample was restricted to children aged 4 years, which may have limited the detection of developmental delays that typically manifest earlier or later in childhood. Third, although confounder selection was informed by DAGs, residual confounding from unmeasured variables such as maternal mental health, maternal obesity and other environmental exposures cannot be ruled out. In addition, including only Qatari nationals may limit the generalisability of the findings to more diverse populations. Also, the study is a retrospective study, which lacks reliance on pre-existing, incomplete, invalid or inconsistently recorded data, leading to high risks of recall, selection and information bias. As this study was based on a previously established retrospective dataset, certain important neonatal conditions, such as sepsis, hospital admissions, nutritional status and socioeconomic factors, which are important determinants of child developmental outcomes, were not systematically available and therefore could not be accounted for in the analysis. Finally, the sample size in the pre-pregnancy diabetes group was particularly limited, reducing statistical power to detect associations in adjusted analyses. More recent randomised controlled trials (RCTs) are needed.

While this study focused on autism, speech delay and motor delays, other neurodevelopmental domains such as general intellectual ability, attention, executive functions and memory were not assessed. These higher-order functions typically require formal neuropsychological testing and may emerge later in childhood. Future longitudinal studies with standardised cognitive and behavioural assessments are needed to evaluate the full neurodevelopmental spectrum associated with maternal diabetes. Also, exclusion of preterm infants creates a bias since some of the effects of maternal diabetes could be mediated through prematurity. Exclusion of those with hypoxia might be removing those who probably had the effect of maternal DM. This is a potential bias that should be noted.

### Implications and future research

Despite non-significant associations between gestational diabetes and neurodevelopmental impairments in children in our study, the trends support ongoing surveillance and early screening in children of mothers with gestational diabetes. Future studies with larger sample sizes and longer follow-up should be undertaken to rigorously establish and reaffirm the effects/no effects and associations/no associations of DM on neurodevelopmental outcomes that may have been missed by this study, which was limited by design and other factors. The method of conception, spontaneous versus in-vitro fertilisation, was not investigated in this study, so the impact of that on neurodevelopmental outcomes is a subject for future studies.

## Conclusion

In conclusion, no significant associations were found between gestational diabetes and early childhood neurodevelopmental delays after adjustment. However, further research studies with a large sample size are needed to assess long-term outcomes in gestational and pre-pregnancy diabetes.

## Supplementary material

10.1136/bmjpo-2025-004232 online supplemental file 1

## Data Availability

Data are available upon reasonable request.
