# Supplementary material for: Neurodevelopmental outcomes of children born to diabetic mothers in the Qatari population: a retrospective study
Source: BMJ Paediatr Open. 2026 May 22;10(1):e004232. doi: 10.1136/bmjpo-2025-004232 (PMC13202112; doi:10.1136/bmjpo-2025-004232)
Supplement: online supplemental file 1 [file bmjpo-10-1-s001.docx]

**Supplementary Tables**

**Supplementary Table 4:** Multivariable logistic regression analysis of autism risk among children born to mothers with diabetes.

| Autism | Odds ratio | 95% Conf. interval | P>z |
| --- | --- | --- | --- |
| Diabetes group |  |  |  |
| GDM | 1.58 | 0.94 - 2.66 | 0.08 |
| Pre-pregnancy DM | 0.46 | 0.06 - 3.52 | 0.45 |
| Maternal age in years | 1.02 | 0.98 -1.06 | 0.35 |
| Gestational age in weeks | 0.94 | 0.77 - 1.15 | 0.55 |
| Sex |  |  |  |
| Male | 2.22 | 1.33 - 3.72 | 0.002 |

**Supplementary Table 5:** Multivariable logistic regression analysis of speech delay risk among children born to mothers with diabetes.

| **Speech delay** | **Odds ratio** | **95% conf. interval** | **P>z** |
| --- | --- | --- | --- |
| **Diabetes group** |  |  |  |
| GDM | 1.39 | 0.97 - 2.00 | 0.07 |
| pre-pregnancy DM | 1.45 | 0.62 - 3.39 | 0.39 |
| **Maternal age in years** | 0.99 | 0.97 - 1.03 | 0.90 |
| **Gestational age in weeks** | 0.90 | 0.79 - 1.03 | 0.13 |
| **sex** |  |  |  |
| Male | 2.09 | 1.49 - 2.94 | <0.001 |

**Supplementary Table 6:** Multivariable logistic regression analysis of gross motor delay risk among children born to mothers with diabetes.

| **Motor delay** | **Odds ratio** | | **95% conf. interval** | **P>z** | |
| --- | --- | --- | --- | --- | --- |
| **Diabetes group** |  |  | | |  |
| GDM | 1.33 | 0.68 -2.59 | | | 0.4 |
| pre-pregnancy DM | 1.2 | 0.26 - 5.63 | | | 0.82 |
| **Maternal age in years** | 1.06 | 1.00 - 1.11 | | | 0.04 |
| **Gestational age in weeks** | 0.97 | 0.76 - 1.24 | | | 0.82 |
| **Sex** |  |  | | |  |
| Male | 1.14 | 0.63- 2.07 | | | 0.67 |

**Supplementary Table 7**: Multivariable logistic regression analysis of fine motor delay risk among children born to mothers with diabetes.

| Fine motor delay | Odds ratio | 95% conf. interval | P>z |
| --- | --- | --- | --- |
| Gestational group |  |  |  |
| GDM | 1.44 | 0.74 - 2.79 | 0.28 |
| pre-pregnancy DM | 1.01 | 0.21 - 4.77 | 0.99 |
| Maternal age in years | 1.06 | 1.01 - 1.12 | 0.02 |
| Gestational age in weeks | 0.87 | 0.68 - 1.11 | 0.27 |
| Sex |  |  |  |
| Male | 1.3 | 0.71 - 2.40 | 0.39 |
