# Supplementary material for: Neurodevelopmental outcomes of children born to diabetic mothers in the Qatari population: a retrospective study
Source: BMJ Paediatr Open. 2026 May 22;10(1):e004232. doi: 10.1136/bmjpo-2025-004232 (PMC13202112; doi:10.1136/bmjpo-2025-004232)
Supplement: Supplementary file 2 [file bmjpo-2025-004232.reviewer_comments.pdf]

PEER REVIEW HISTORY

BMJ Paediatrics Open publishes all reviews undertaken for accepted manuscripts. Reviewers are asked to complete a checklist review form and are provided with free text boxes to elaborate on their assessment. These free text comments are reproduced below.

ARTICLE DETAILS

**TITLE (PROVISIONAL)**	Neurodevelopmental Outcomes of Children Born to Diabetic Mothers in the Qatari Population: A Cross-Sectional Study.
**AUTHORS**	Masri, Razan M.<br>Bayoumi, Mohammad A. A.<br>Amin, Rasha I<br>Alsharif, Hafsa O. A.<br>Musa, Amna<br>Mudrek, Ahmad S.<br>Selim, Basma A. M. R.<br>Chandra, Prem<br>Elshaar, Samah

VERSION 1 – REVIEW

**REVIEWER NAME**	Magai, Dorcas
**REVIEWER AFFILIATION**	University of Liverpool, Public Health, Policy and Systems
**REVIEWER CONFLICT OF INTEREST**	No Conflict of Interest
**AI DISCLOSURE**	No, I have not used AI
**DATE REVIEW RETURNED**	08-Dec-2025

**GENERAL COMMENTS**	The study titled “Neurodevelopmental Outcomes of Infants of Diabetic Mothers in the Qatari Population” examined the association between maternal diabetes and neurodevelopmental outcomes in children at 4 years of age. The study reports that children of mothers with maternal diabetes were at an increased risk of poorer neurodevelopmental outcomes, although these associations were not statistically significant after considering the effects of confounding factors. The study provides important insights into the importance of prenatal care and glycaemic monitoring during pregnancy.<br>The following comments are opportunities to strengthen the manuscript:<br>Abstract<br>Line 83: GDM should be written in expanded form.<br>Introduction<br>The introduction is generally well written but oversimplified. The introduction section would benefit from providing information about how child neurodevelopment is shaped by various factors and how gestational diabetes is likely to influence child neurodevelopment. This information would provide the reader with a background of the research and link with the results and discussion sections.<br><br>Line 117: Reference #3 does not support the information cited. The Al-Wardat et al (2024) paper is about the prevalence of ADHD, not diabetes mellitus. The authors should ensure all references are correctly cited.<br>Methods

	Line 161: It is unclear how the authors defined the term infants. This should be stated explicitly in the paper.<br>Line 167-171: Is this exclusion criteria for the whole sample, or is it only for the unexposed group?<br>Study variables should be clearly defined and authors should state how they were objectively measured to ensure replicability of the study findings.<br>Line 177: How were speech/motor delays defined in the institutional and national guidelines? Line 178: What standardised assessment tools were used? A description of these should be provided.<br>-how was GMD, pregnancy MD diagnosed<br>-how was gestational age calculated?<br>-how was Neonatal hypoglycemia and HbA1C defined?<br>181: Were other important neonatal predictors of child developmental delays e.g. other neonatal conditions such as sepsis, and other factors including hospital admissions, nutrition status, and socioeconomic status, considered? These should be included in the analysis.<br>Results<br>Lines 249, 269, and 315, it is unclear what potential confounders were accounted for (maternal age and gestational age?). These should be explicitly stated so it is clearer to the reader.<br>Discussion.<br>The discussion can be further improved by providing explanation to the findings. E.g. Lines 378/379: what confounders were adjusted for, and how do they influence ASD risk in children?<br>Line 353: the prevalence of gestational diabetes reported by the Gulf Cooperation is captured as 27.5% which is higher than the findings of the current study but contradicts the statement: Compared to the MENA region (13%), the Gulf Cooperation Council (GCC) (27.5%)22, and the global rate (14%)23, our rate is elevated.<br>Line 402: The authors claim that “the absence of statistically significant associations between gestational diabetes and neurodevelopmental outcomes in our study may be explained by the relatively mild nature of GDM in our cohort” yet the results do not provide these associations based on the severity of GDM. The authors should add a sensitivity analysis with the level of GDM severity to substantiate this claim.<br>The clinical implications of these findings should be elaborated.<br><br>Minor comment:<br>Add the expanded forms of all abbreviated words e.g GDM, DM, HbA1C to the tables captions.

**REVIEWER NAME**	Nampijja, Margaret
**REVIEWER AFFILIATION**	African Population and Health Research Center, Human Development
**REVIEWER CONFLICT OF INTEREST**	No Conflict of Interest
**AI DISCLOSURE**	No, I have not used AI
**DATE REVIEW RETURNED**	03-Jan-2026

**GENERAL COMMENTS**	This is a very interesting manuscript focusing on an important yet under studied topic i.e. developmental outcomes of maternal diabetes in the Qatar population. The paper presents findings of an observational, cross-sectional, retrospective study. The article including study objectives, design and findings are well described and presented. There are however, a few issues highlighted below

	that should to be addressed to improve the quality and strength of the paper. These are:<br><br>Page 2: line 3: Title: It would be useful to indicate the study design in the title e.g. 'Neurodevelopmental Outcomes of Infants of Diabetic Mothers in the Qatari Population: a cross-sectional study.'<br><br>Page 3: line 45 - summary box: A word is missing after support. Might be 'and'.<br><br>Keywords: It is not clear why 'qualitative' is among the key words listed yet this an exclusively quantitative study. I suggest this should be deleted and replaced with quantitative.<br><br>Page 4: line 81: Neurodevelopmental outcomes were assessed in 4 year olds; hence the use of the word infants to refer to the children is not correct. It should be replaced including in the title with children (early childhood). Infancy usually refers to the first year of life or at most up to 2 years.<br><br>It would be useful for the authors to clarify on the rationale for the selection of the neurodevelopmental disorders that were examined given that there are several other aspects that might also be at risk including general intellectual ability, attention, executive functions, memory etc.<br>Furthermore, ASD is a syndrome, for which identification of those with the condition must satisfy the diagnostic criteria. Yet it is possible that some of the participants were not typically found to be with ASD but had isolated behavioral abnormalities. The authors should provide more information on this.<br>Page 5: line 134: This study aims .... should be in the past tense. Similarly the earlier sentence ...' the study seeks' should be in the past tense.<br>Page 6: study variables: Line 172-183.<br>It is not clear which variables (data) were retrieved from medical records i.e as secondary data and which were collected through primary data or direct participant assessments in this study. This should be explicit.<br><br>Page 7: Ethical considerations: line 197. The authors should clarify whether the participants (women and their children) were engaged at any point through observation, interviews or assessments during this study; in which case their consent was required. If they were not directly involved and all information was from the records, this needs to be clear here, and hence the reason for not seeking their consent.<br><br>Apart from timing of the of the diabetes, it would also be useful to assess the severity of the diabetes and how this was associated with the neurodevelopmental outcomes.<br><br>Also, were there any interactions between for example GDM, maternal age and child's gender?<br>Were any of the children found to have multiple adverse outcomes (ASD, 7Speech delay, Gross and fine motor delay)?<br><br>Apgar score was treated as a potential confounding variable; however, it is possible that it might be a mediating factor for pre-pregnancy and GDM, implying that adjusting for it would be adjusting these exposures for themselves, and hence diminishing the true effects of these exposures. It would be useful to fist look at

	the association between diabetes and Apgar score, before it is treated as a confounder. Similar to birth length.<br>The authors should list the potential cofounders that were adjusted for in each of the analyses. Are these the ones listed in the Tables, or were there others? This is not clear.<br>Limitation: The authors list potential limitations of the study, it is also important to acknowledge the limitations of retrospective study design and use of secondary data (medical records) including data quality, validity, accuracy etc.

**REVIEWER NAME**	Harron, Katie
**REVIEWER AFFILIATION**	UCL Great Ormond Street Institute of Child Health
**REVIEWER CONFLICT OF INTEREST**	No Conflict of Interest
**AI DISCLOSURE**	No, I have not used AI
**DATE REVIEW RETURNED**	20-Jan-2026

**GENERAL COMMENTS**	Title: need to state study design.<br><br>Abstract methods: It would be helpful to state what the data source is here. Is this administrative data? Hospital records? This is important so that readers understand the study design.<br><br>Abstract results: please state absolute results as well as the odds ratios – i.e. how many children had autism or speech delay?<br><br>Why were data from 2017 used? Follow up would have ended in 2021 so these data are already 5 years old – are there no more recent data?<br><br>The background section states that the aim was to identify prognostic factors (given the rising prevalence of GDM). This suggests prognostic factors for GDM (not autism etc) and so doesn't seem to marry up with what the paper actually sets out to do, which is looking at long-term outcomes. Some rewording here would be helpful to ensure that there is consistency in terms of the aim of the study.<br><br>The methods section states that approximately three controls were chosen for each case. Can you provide more detail on how these controls were chosen, and why a full cohort could not be analysed?<br><br>Were the assessments for ASD, SD etc given to all families irrespective of the diagnosis of GDM? i.e. is this routine practice and would the control families have been equally likely to have these assessments carried out?<br><br>The methods states that women giving birth only in WWRC at HMC were included, but the results state the population born to Qatari mothers recorded in Qatar. Could this be clarified?<br><br>Table 1 – I suggest making the distinction between maternal variables and baby variables more clear here (through appropriate ordering of the variables).<br>Ordering of figures needs to be checked – the first one referenced within the text is Figure 3.<br><br>Figure 3- there is no direct line between maternal age and

	gestational age (nor birth weight). Can this be justified given what we know about these relationships? How was this DAG developed? This is crucial to this paper, to ensure that the correct variables were considered in the relationship between diabetes and outcomes.<br><br>It is not appropriate to interpret the association between covariates and outcome (e.g. on page 10) from multivariable models unless these variables were the primary exposure of interest. This is because the other covariates in the model were selected based on the DAG which had GDM as the exposure (not, for example, newborn gender). I suggest removing this section (and the associated inferences in the discussion and the abstract).<br><br>Appendix tables – need to provide units e.g. years for maternal age and weeks for gestational age.<br><br>I'm surprised that the associations with the outcomes were modelled for pre-pregnancy DM, given the very low numbers of children in this group with the outcomes of interest. The confidence intervals are wide. Can the authors comment on the utility of this analysis?<br><br>What was the level of missing data and how were missing data dealt with?<br><br>The discussion on page 19 (paragraph starting "Additionally, the associations…") talks about key confounding factors but then goes on to discuss male sex. This is not a confounder as it is not related to the exposure.<br><br>The authors state that the STROBE guidelines were used but there were several aspects missing so I would suggest that the authors run through the checklist again.<br><br>Figure 1: It would be more helpful to present % here rather than the absolute numbers<br><br>Figure 2: suggest move this to the appendix as it is not the primary exposure of interest

VERSION 1 – AUTHOR RESPONSE

Reviewer: 1
Dr. Dorcas Magai, University of Liverpool
Comments to the Author
The study titled "Neurodevelopmental Outcomes of Infants of Diabetic Mothers in the Qatari Population" examined the association between maternal diabetes and neurodevelopmental outcomes in children at 4 years of age. The study reports that children of mothers with maternal diabetes were at an increased risk of poorer neurodevelopmental outcomes, although these associations were not statistically significant after considering the effects of confounding factors. The study provides important insights into the importance of prenatal care and glycaemic monitoring during pregnancy. The following comments are opportunities to strengthen the manuscript:
Abstract
Line 83: GDM should be written in expanded form.
It has been added.
Introduction
The introduction is generally well written but oversimplified. The introduction section would benefit from providing information about how child neurodevelopment is shaped by various factors and how

gestational diabetes is likely to influence child neurodevelopment. This information would provide the reader with a background of the research and link with the results and discussion sections.
Thank you very much for your valuable comment. The following paragraph has been added to the introduction:
Many factors, including genetic and environmental factors, influence the structural and functional development of the brain. Environmental factors include drugs, irradiation, chemicals, infections, malnutrition, antenatal stress, maternal separation, and metabolic derangements3,5. Intrauterine hyperglycemia resulting from GDM is one of these sub-optimal developmental factors. These factors can negatively impact any stage of neurodevelopment, including neurogenesis, neuronal differentiation, neuronal migration, neuronal cell apoptosis, axonal connections, and synaptogenesis. These negative impacts can be through direct effects and/or indirectly through epigenetic dysregulation, and might lead to irreversible damage to the brain3. GDM is associated with disturbances in insulin metabolism and insulin signalling, the inflammatory process, oxidative stress, and epigenetic modification that might lead to abnormal neurodevelopment and neuropsychiatric disorders1,3.
Line 117: Reference #3 does not support the information cited. The Al-Wardat et al (2024) paper is about the prevalence of ADHD, not diabetes mellitus. The authors should ensure all references are correctly cited.
Thank you very much. The sentence has been removed, and the reference has been replaced.
Methods
Line 161: It is unclear how the authors defined the term infants. This should be stated explicitly in the paper.
Term infants were those born between 37 weeks, 0 days and 41 weeks, 6 days of gestation. This has been added to the manuscript.
Line 167-171: Is this exclusion criteria for the whole sample, or is it only for the unexposed group?
It is from the whole sample. It has been added to the manuscript.
Study variables should be clearly defined and authors should state how they were objectively measured to ensure replicability of the study findings.
The following paragraphs were added/edited in the methods section:
Speech delay was defined as expressive language abilities that are substantially and quantifiably below age expectations, resulting in functional limitations in communication, social participation, academic achievement, or occupational performance. Motor delay was defined as the gross or fine motor skill abilities that are substantially and quantifiably below age expectation, resulting in functional limitation with activities of daily living, academic productivity, leisure and play.
Autism Spectrum Disorder (ASD) was assessed and diagnosed using the Autism Diagnostic Observation Schedule, Second Edition (ADOS-2). It is a semi-structured, standardised direct observational assessment tool that evaluates social communication, social interaction, play, and restricted/repetitive behaviours consistent with ASD. Speech delay was assessed and diagnosed with the help of a speech and language therapist using the Preschool Language Scale (PLS-5). It is a standardised developmental language test assessing receptive and expressive language skills in infants and young children through structured play and interaction. Motor delay was assessed and diagnosed with the help of the physiotherapist and/or occupational therapist using assessment tools like the Bayley Scales of Infant and Toddler Development (Bayley-4), or Peabody Developmental Motor Scales (PDMS-2), which both are standardized assessment tools for motor development in young children, including reflexes, balance, grasping, and visual-motor integration.
Other maternal variables collected from the electronic medical records included maternal age, gestational age, Body Mass Index (BMI), periconceptional and most recent Glycosylated Haemoglobin (HbA1c%) in pregnancy, timing of GDM diagnosis, type of GDM therapy, placental weight, and other maternal pregnancy complications.
Line 177: How were speech/motor delays defined in the institutional and national guidelines?
Thank you very much for your valuable comment. We have added the following to the methods section in the manuscript.

Speech delay was defined as expressive language abilities that are substantially and quantifiably below age expectations, resulting in functional limitations in communication, social participation, academic achievement, or occupational performance. Motor delay was defined as the gross or fine motor skill abilities that are substantially and quantifiably below age expectation, resulting in functional limitation with activities of daily living, academic productivity, leisure and play.
Line 178: What standardised assessment tools were used? A description of these should be provided.
Thank you very much for your valuable comment. We have added the following to the methods section in the manuscript.
Autism Spectrum Disorder (ASD) was assessed and diagnosed using the Autism Diagnostic Observation Schedule, Second Edition (ADOS-2). It is a semi-structured, standardised direct observational assessment tool that evaluates social communication, social interaction, play, and restricted/repetitive behaviours consistent with ASD. Speech delay was assessed and diagnosed with the help of a speech and language therapist using the Preschool Language Scale (PLS-5). It is a standardised developmental language test assessing receptive and expressive language skills in infants and young children through structured play and interaction. Motor delay was assessed and diagnosed with the help of the physiotherapist and/or occupational therapist using assessment tools like the Bayley Scales of Infant and Toddler Development (Bayley-4), or Peabody Developmental Motor Scales (PDMS-2), which both are standardized assessment tools for motor development in young children, including reflexes, balance, grasping, and visual-motor integration.
-how was GMD, pregnancy MD diagnosed
The following paragrapgh has been added to the methods section:
All the medical institutions in Qatar screen all pregnant ladies for diabetes at the first antenatal care visit. Accordingly, pregnant ladies are classified. That national screening is based on the 2013-WHO Criteria which states that GDM should be diagnosed at any time in pregnancy if one or more of the following criteria are met: fasting plasma glucose 5.1-6.9 mmol/l (92 -125 mg/dl), 1-hour plasma glucose ≥ 10.0 mmol/l (180 mg/dl) following a 75g oral glucose load, or 2-hour plasma glucose 8.5-11.0 mmol/l (153 -199 mg/dl) following a 75g oral glucose load4,5. Pre-pregnancy DM was defined by either type 1 or type 2 DM before the index pregnancy.
-how was gestational age calculated?
The following paragraph has been added:
Gestational age was mainly calculated using the Last Menstrual Period (LMP), calculated from the first day of the last normal menstrual period. This assumes a 28-day cycle, with ovulation occurring on day 14. Also, an early obstetric ultrasound done in the first trimester (up to 13 6/7 weeks) was used to determine accurate gestational age using measurements like the Crown-Rump Length (CRL). In cases of In Vitro Fertilisation (IVF), gestational age was calculated by adding 14 days to the date of oocyte retrieval or 3/5-day embryo transfer.
-how was Neonatal hypoglycemia and HbA1C defined?
The following paragrapgh has been added to the manuscript:
We define neonatal hypoglycemia based on the plasma glucose level for those who are less than 48 hours of life with plasma glucose levels <50 mg/dL (2.8 mmol/L), and for those who are greater than 48 hours of life with plasma glucose levels <60 mg/dL (3.3 mmol/L). The last HbA1C% (Glycated Hemoglobin) was checked before delivery in women with GDM and those who had pre-pregnancy DM to get an idea about glycemic control in the preceding 3 months.
181: Were other important neonatal predictors of child developmental delays e.g. other neonatal conditions such as sepsis, and other factors including hospital admissions, nutrition status, and socioeconomic status, considered? These should be included in the analysis.
Thank you for highlighting this point. We acknowledge that neonatal conditions such as sepsis, hospital admissions, nutritional status, and socioeconomic factors are important predictors of child developmental outcomes. However, this analysis was based on a previously established retrospective dataset of Qatari mothers with gestational or pre-pregnancy diabetes mellitus who delivered at the Women’s Wellness and Research Center, Hamad Medical Corporation, and these variables were not systematically available as the dataset was originally collected for a different research purpose. This

limitation has now been added to the manuscript.
Results
Lines 249, 269, and 315, it is unclear what potential confounders were accounted for (maternal age and gestational age?). These should be explicitly stated so it is clearer to the reader.
Thank you for this comment. We acknowledge the importance of clearly identifying the adjusted confounders. These variables were originally specified in the footnote of Table 3, indicating that all four outcome variables were adjusted for the minimal adjustment set of maternal age, gestational age, and child sex. To further enhance clarity for the reader, these confounders have now also been explicitly stated in the Results section.
Discussion.
The discussion can be further improved by providing explanation to the findings. E.g. Lines 378/379: what confounders were adjusted for, and how do they influence ASD risk in children?
Thank you for this important point. We have now expanded the discussion section to clearly state the confounders adjusted for and to explain their potential role in attenuating the observed association. In addition, three references have been added to this section to support the proposed explanation.
Line 353: the prevalence of gestational diabetes reported by the Gulf Cooperation is captured as 27.5% which is higher than the findings of the current study but contradicts the statement: Compared to the MENA region (13%), the Gulf Cooperation Council (GCC) (27.5%)22, and the global rate (14%)23, our rate is elevated.
The second sentence has been corrected to be:
The prevalence of gestational diabetes in the MENA region was 13%, in the Gulf Cooperation Council (GCC) 27.5%22, and globally 14%23.
Line 402: The authors claim that “the absence of statistically significant associations between gestational diabetes and neurodevelopmental outcomes in our study may be explained by the relatively mild nature of GDM in our cohort” yet the results do not provide these associations based on the severity of GDM. The authors should add a sensitivity analysis with the level of GDM severity to substantiate this claim.
Thank you for this important and constructive comment. In response, we have conducted additional sensitivity analyses stratifying gestational diabetes by treatment modality as a proxy for disease severity. The following changes have been made to address this concern:
1. A description of the severity-based sensitivity analysis has been added to the Methods section.
2. A new paragraph summarizing the results of the sensitivity analyses has been added to the Results section.
3. A new table presenting detailed results of the severity-based sensitivity analyses has been added to the Appendix (Table 8).
4. The corresponding sentence in the Discussion has been revised to reflect these findings and to align the interpretation with the new analyses.
The sensitivity analyses demonstrated consistent null associations across diet-controlled and medication-treated GDM, supporting the interpretation that the predominantly mild GDM profile of the cohort may have contributed to the observed null findings.
The clinical implications of these findings should be elaborated.
The following paragragh has been added to the discussion section:
The absence of statistically significant associations between gestational diabetes and neurodevelopmental outcomes may be partly attributable to the predominantly mild GDM profile of the cohort, as sensitivity analyses stratified by disease severity demonstrated no associations across diet-controlled and medication-treated GDM.
Minor comment:
Add the expanded forms of all abbreviated words e.g GDM, DM, HbA1C to the tables captions.
It has been added.
Reviewer: 2
Margaret Nampiija
Comments to the Author

This is a very interesting manuscript focusing on an important yet under studied topic i.e. developmental outcomes of maternal diabetes in the Qatar population. The paper presents findings of an observational, cross-sectional, retrospective study. The article including study objectives, design and findings are well described and presented. There are however, a few issues highlighted below that should to be addressed to improve the quality and strength of the paper. These are:

Page 2: line 3: Title: It would be useful to indicate the study design in the title e.g. 'Neurodevelopmental Outcomes of Infants of Diabetic Mothers in the Qatari Population: a cross-sectional study.'
The study design has been added to the title.
Page 3: line 45 - summary box: A word is missing after support. Might be 'and'.
It has been added.
Keywords: It is not clear why 'qualitative' is among the key words listed yet this an exclusively quantitative study. I suggest this should be deleted and replaced with quantitative.
It has been replaced.
Page 4: line 81: Neurodevelopmental outcomes were assessed in 4 year olds; hence the use of the word infants to refer to the children is not correct. It should be replaced including in the title with children (early childhood). Infancy usually refers to the first year of life or at most up to 2 years.
It has been corrected in the title and the manuscript.
It would be useful for the authors to clarify on the rationale for the selection of the neurodevelopmental disorders that were examined given that there are several other aspects that might also be at risk including general intellectual ability, attention, executive functions, memory etc.
Thank you very much for your valuable comments.
The selection of autism spectrum disorder, speech delay, and gross and fine motor delays was guided by a couple of methodological and clinical considerations.
First, these outcomes represent core, early-emerging neurodevelopmental domains that are routinely assessed and reliably documented in early childhood clinical practice, particularly at the age of 4 years, which was the standardised follow-up point in our cohort. In contrast, higher-order cognitive domains such as general intellectual ability, attention, executive functions, and memory typically require formal neuropsychological testing, which is not systematically performed or consistently recorded in routine clinical care at this age in our setting.
Secondly, the retrospective design and reliance on electronic medical records necessitated the selection of outcomes with clear diagnostic criteria, standardised clinical assessments, and high documentation reliability. Neurocognitive constructs such as executive function and memory are more vulnerable to misclassification and under-ascertainment in retrospective datasets, particularly in the absence of uniform testing protocols.
We agree that other neurodevelopmental domains, including intellectual functioning and attention, may also be affected by intrauterine exposure to maternal diabetes. This limitation has been acknowledged, and we emphasise that future longitudinal studies incorporating comprehensive neuropsychological assessments across later childhood are needed to capture these higher-order outcomes more fully.
We have added the following paragraph to the manuscript:
While this study focused on autism, speech delay, and motor delays, other neurodevelopmental domains such as general intellectual ability, attention, executive functions, and memory were not assessed. These higher-order functions typically require formal neuropsychological testing and may emerge later in childhood. Future longitudinal studies with standardised cognitive and behavioural assessments are needed to evaluate the full neurodevelopmental spectrum associated with maternal diabetes.
Furthermore, ASD is a syndrome, for which identification of those with the condition must satisfy the diagnostic criteria. Yet it is possible that some of the participants were not typically found to be with ASD but had isolated behavioral abnormalities. The authors should provide more information on this.
In our study, autism spectrum disorder was defined based on a formal clinical diagnosis documented

in the medical record, made by a qualified developmental paediatrician, according to DSM-5 diagnostic criteria in place at the time of assessment. Only children with a documented ASD diagnosis were classified as having autism in the analysis.

Children with isolated behavioural concerns who did not meet full diagnostic criteria for ASD were not classified as ASD cases and were instead included in the non-ASD comparison group. Thus, the ASD outcome reflects categorical clinical diagnoses rather than subclinical autistic traits or screening-level behavioural abnormalities.

Page 5: line 134: This study aims .... should be in the past tense. Similarly the earlier sentence ...' the study seeks' should be in the past tense.

It has been corrected.

Page 6: study variables: Line 172-183.

It is not clear which variables (data) were retrieved from medical records i.e as secondary data and which were collected through primary data or direct participant assessments in this study. This should be explicit.

It has been rewritten in detail in the study variables parts of the methods section.

Page 7: Ethical considerations: line 197. The authors should clarify whether the participants (women and their children) were engaged at any point through observation, interviews or assessments during this study; in which case their consent was required. If they were not directly involved and all information was from the records, this needs to be clear here, and hence the reason for not seeking their consent.

The following sentences have been added:

There was no consent for the publication of this research because there was no identity disclosure was made in this study. It is a retrospective data review study. Consent to participate was waived by the IRB at HMC according to the national regulations, as the study is a retrospective observational study.

Apart from timing of the of the diabetes, it would also be useful to assess the severity of the diabetes and how this was associated with the neurodevelopmental outcomes.

Thank you for this valuable comment. In response, we have conducted additional sensitivity analyses stratifying gestational diabetes by treatment modality as a proxy for disease severity. The following changes have been made to address this concern:

1. A description of the severity-based sensitivity analysis has been added to the Methods section.
2. A new paragraph summarizing the results of the sensitivity analyses has been added to the Results section.
3. A new table presenting detailed results of the severity-based sensitivity analyses has been added to the Appendix (Table 8).
4. The corresponding sentence in the Discussion has been revised to reflect these findings and to align the interpretation with the new analyses.

Also, were there any interactions between for example GDM, maternal age and child's gender?

Thank you for this important question. to clarify, we formally assessed potential effect modification by including interaction terms between gestational diabetes and key covariates, including maternal age, gestational age, and child sex, in the multivariable logistic regression models and no statistically significant interactions were identified. However, To improve clarity for readers, we have added explicit statements reporting the absence of interaction effects in the Results section.

Were any of the children found to have multiple adverse outcomes (ASD, 7Speech delay, Gross and fine motor delay)?

Thank you for highlighting this important point. Yes, some children experienced more than one adverse neurodevelopmental outcome. However, during our analysis, each neurodevelopmental outcome was analyzed independently as a separate endpoint, allowing children with multiple outcomes to contribute to each relevant outcome-specific analysis. To clarify this approach for readers, we have updated the Methods section accordingly.

Apgar score was treated as a potential confounding variable; however, it is possible that it might be a mediating factor for pre-pregnancy and GDM, implying that adjusting for it would be adjusting these

exposures for themselves, and hence diminishing the true effects of these exposures. It would be useful to fist look at the association between diabetes and Apgar score, before it is treated as a confounder. Similar to birth length.
Thank you for this important comment. To clarify, Apgar score and birth length were considered as potential confounders; however, based on our directed acyclic graph (DAG) and literature search, these variables were identified as mediators on the causal pathway between maternal diabetes and neurodevelopmental, and adjusting for them would therefore represent overadjustment, which goes in line with what you mentioned above. Consequently, they were not included in the multivariable models, which were adjusted for maternal age, gestational age, and child sex as the minimal sufficient adjustment set.
The authors should list the potential cofounders that were adjusted for in each of the analyses. Are these the ones listed in the Tables, or were there others? This is not clear.
We thank the reviewer for this comment and agree that clarity is important. The confounders adjusted for in the multivariable analyses were limited to those listed in the tables. The minimal sufficient adjustment was maternal age, gestational age, and child sex. To further enhance clarity for readers, we have now explicitly stated the adjusted confounders in the Results section.
Limitation: The authors list potential limitations of the study, it is also important to acknowledge the limitations of retrospective study design and use of secondary data (medical records) including data quality, validity, accuracy etc.
The following sentence has been added to the limitations:
Also, the study is a retrospective study, which lacks reliance on pre-existing, incomplete, invalid, or inconsistently recorded data, leading to high risks of recall, selection, and information bias.
Reviewer: 3
Prof. Katie Harron, UCL Great Ormond Street Institute of Child Health
Comments to the Author
Title: need to state study design.
The study design has been added to the title.
Abstract methods: It would be helpful to state what the data source is here. Is this administrative data? Hospital records? This is important so that readers understand the study design.
The following sentence has been added to the abstract methods:
Data for the study were retrieved from electronic medical records.
Abstract results: please state absolute results as well as the odds ratios – i.e. how many children had autism or speech delay?
It has been rephrased with absolute results.
Why were data from 2017 used? Follow up would have ended in 2021 so these data are already 5 years old – are there no more recent data?
This is a retrospective data review study. The approved period by the IRB was from 2017 to 2021. More recent randomised controlled trials (RCTs) are needed. This has been written in the limitations.
The background section states that the aim was to identify prognostic factors (given the rising prevalence of GDM). This suggests prognostic factors for GDM (not autism etc) and so doesn't seem to marry up with what the paper actually sets out to do, which is looking at long-term outcomes. Some rewording here would be helpful to ensure that there is consistency in terms of the aim of the study.
It has been reworded to be:
This study aimed to investigate the long-term outcomes, including certain neurodevelopmental disorders, including autism spectrum disorder (ASD), speech delay (SD), and gross and fine motor delays in children who were born to Qatari women with GDM or pre-pregnancy diabetes.
The methods section states that approximately three controls were chosen for each case. Can you provide more detail on how these controls were chosen, and why a full cohort could not be analysed?
The sentence has been edited as follows:
Controls were all children of the same age, born in 2017 to non-diabetic Qatari mothers. No selection of controls was made.
Were the assessments for ASD, SD etc given to all families irrespective of the diagnosis of GDM? i.e.

is this routine practice and would the control families have been equally likely to have these assessments carried out?
Thank you very much for your comments. The following paragraph has been added to the methods section of the manuscript:
Neurodevelopmental assessments for autism spectrum disorder, speech delay, and motor delay were not routinely performed for all children but were initiated based on standard developmental surveillance and clinical concern. The decision to screen, refer, or assess a child was not influenced by maternal gestational diabetes status. Children in both the GDM and non-GDM groups were followed through the same healthcare system, had access to the same developmental surveillance during well-child visits, and were referred as needed for assessment using the same criteria.
The methods states that women giving birth only in WWRC at HMC were included, but the results state the population born to Qatari mothers recorded in Qatar. Could this be clarified?
The sentence in the results section has been corrected to be: In 2017, there were a total of 17,020 live births in WWRC.
Table 1 – I suggest making the distinction between maternal variables and baby variables more clear here (through appropriate ordering of the variables).
It has been rearranged.
Ordering of figures needs to be check ed – the first one referenced within the text is Figure 3.
It has been adjusted.
Figure 3- there is no direct line between maternal age and gestational age (nor birth weight). Can this be justified given what we know about these relationships? How was this DAG developed? This is crucial to this paper, to ensure that the correct variables were considered in the relationship between diabetes and outcomes.
We thank you for this important comment. The directed acyclic graph (DAG) was initially developed based on existing literature and clinical knowledge of the relationships between maternal diabetes, perinatal factors, and neurodevelopmental outcomes. Following your respected suggestion, we carefully revisited all assumed causal relationships and updated the DAG accordingly. The revised DAG (now in Figure 1) has replaced the original figure in the manuscript, and all relationships have been re-evaluated to ensure alignment with current knowledge and appropriate confounder selection.
It is not appropriate to interpret the association between covariates and outcome (e.g. on page 10) from multivariable models unless these variables were the primary exposure of interest. This is because the other covariates in the model were selected based on the DAG which had GDM as the exposure (not, for example, newborn gender). I suggest removing this section (and the associated inferences in the discussion and the abstract).
We thank you for this important methodological clarification and agree that interpretation of associations between covariates and outcomes from multivariable models is not appropriate when these variables were included solely for confounding control. Accordingly, we have removed the related inferences from the Results, Discussion, and Abstract.
However, we would like to note that unadjusted (crude) associations are retained and presented descriptively, as they may be informative for hypothesis generation and could motivate future studies in which these variables are examined as primary exposures. All related text has been revised to ensure that inference is based exclusively on models evaluating gestational diabetes as the exposure of interest. These changes can be found on pages 3, 9, 11, 13, and 15 of the revised manuscript.
Appendix tables – need to provide units e.g. years for maternal age and weeks for gestational age.
It has been added.
I'm surprised that the associations with the outcomes were modelled for pre-pregnancy DM, given the very low numbers of children in this group with the outcomes of interest. The confidence intervals are wide. Can the authors comment on the utility of this analysis?
We thank the reviewer for this important observation. We acknowledge that the number of children exposed to pre-pregnancy diabetes was small, resulting in limited statistical power and wide confidence intervals. Nevertheless, we believe it is important to present analyses of pre-pregnancy diabetes separately from gestational diabetes, as existing literature indicates that these conditions

differ in pathogenesis, severity, and associated maternal and fetal complications. The analysis of pre-pregnancy diabetes was therefore included to provide descriptive estimates and contextual insight rather than definitive inference. We agree that these findings should be interpreted with caution, and this limitation has been clearly stated in the Discussion section.
What was the level of missing data and how were missing data dealt with?
We thank the reviewer for highlighting this important point. We acknowledge that the initial version of the manuscript did not sufficiently describe the extent of missing data. To address this, we have now added a detailed table summarizing the level of missingness for all study variables in the Appendix (Table 9), and we have included a corresponding description in the Methods section.
Overall, missing data varied across variables, with minimal missingness for the primary exposure, birth measures, and key covariates included in the main analyses such as gestational age, maternal age, and sex; moderate missingness for neurodevelopmental outcomes and Apgar scores; and substantial missingness for metabolic and treatment-related variables. Primary analyses were conducted using complete-case analysis, as missingness was minimal for the exposure, outcomes, and covariates in the minimal adjustment set. Variables with substantial missingness were not included in the main multivariable models and were used only in secondary or sensitivity analyses.
The discussion on page 19 (paragraph starting "Additionally, the associations…") talks about key confounding factors but then goes on to discuss male sex. This is not a confounder as it is not related to the exposure.
We thank the reviewer for this important clarification. We agree that child sex is not a confounder in the association between gestational diabetes and neurodevelopmental outcomes, as it is not causally related to the exposure. We have therefore revised the Discussion section to remove references to child sex as a confounding factor and to clarify its role as a potential effect modifier.
The authors state that the STROBE guidelines were used but there were several aspects missing so I would suggest that the authors run through the checklist again.
It has been completed.
Figure 1: It would be more helpful to present % here rather than the absolute numbers
We thank you for this helpful suggestion. Figure 1 has been revised to present percentages rather than absolute numbers, and the updated figure has replaced the original version in the manuscript (now Figure 3).
Figure 2: suggest move this to the appendix as it is not the primary exposure of interest
We thank the reviewer for this suggestion. Figure 2 has been moved to the Appendix.
Reviewer: 1
If you have selected 'Yes' above, please provide details of any competing interests.: N/A

Reviewer: 2
If you have selected 'Yes' above, please provide details of any competing interests.: Not applicable

Reviewer: 3
If you have selected 'Yes' above, please provide details of any competing interests.: na

VERSION 2 – REVIEW

**REVIEWER NAME**	Magai, Dorcas
**REVIEWER AFFILIATION**	University of Liverpool, Public Health, Policy and Systems
**REVIEWER CONFLICT OF INTEREST**	No Conflict of Interest
**AI DISCLOSURE**	No, I have not used AI
**DATE REVIEW RETURNED**	23-Mar-2026

**GENERAL COMMENTS**	Many thanks to the authors for revising the manuscript and for their careful consideration of my previous comments. Please see below additional minor comment:

	The last paragraph under the Introduction section would be more appropriately placed under the Limitation section.

**REVIEWER NAME**	Nampijja, Margaret
**REVIEWER AFFILIATION**	African Population and Health Research Center, Human Development
**REVIEWER CONFLICT OF INTEREST**	No Conflict of Interest
**AI DISCLOSURE**	No, I have not used AI
**DATE REVIEW RETURNED**	26-Mar-2026

**GENERAL COMMENTS**	Most of the previous issues have been addressed, and the manuscript has improved. However, there are a few other issues that need to be addressed.<br>The manuscript has improved; however, a few issues need to be addressed.<br>Line 158: Exclusion of preterm infants creates a bias since some of the effects of maternal diabetes could be mediated through prematurity. This potential limitation needs to be acknowledged under limitations.<br>Line 163: Pregnant ladies- women would sound more appropriate.<br>166: The fasting blood sugar range indicated for diagnosing of DM is incorrect. It should be a fasting glucose result 5.1mmol/L or greater.<br>174-175: Provide information on how many children were from in vitro fertilization.<br>State clearly whether any data that was used in this study was primary data or if all data were obtained from records.<br>Line 197 and 229: Glycated and glycosylated - choose one of the two terms for consistency.<br>Line 200. Clarify what is meant by " No selection of controls was made.'<br>Line 234: 'Data analysis and collection were kept in an excel sheet on password secured computers'. The sentence should be revised; does not sound correct.<br>Line 264: The statement " There was no There was no consent for the publication of this research because there was no identity disclosure was made in this study. ' is grammatically not reading right.<br>Also note that deidentifying data in a publication does not justify a waiver for consenting. In a different place, a different reason was given for not obtaining participant consent. The justification for not having obtained participant consent should be consistent. The key justification here could be the use of secondary data from medical records.<br>Table 1A; The total number N= 5141 should be at the end of the Table heading. In the table itself the statistics in and outside the brackets should be defines e.g. n(%).<br>439: Provide a description of the MENA region for more context.<br>441: the sentence "....can be attributed to a multifactorial nature”..... sounds incomplete. It should be revised.<br>443: It is not likely that longitudinal studies can mitigate the incidence of diabetes. This sentence should be revised.<br>503: ...higher Apgar scores may have had a protective effect.. this is misleading since as the author later indicates, Apgar score is a reflection of the wellbeing of the newborn. In itself, it is not an exposure. The sentence should be rephrased.<br>506: Moreover neurodevelopmental outcomes differed by sex with

	higher prevalence observed among males. Outcomes could be replaced with impairments.<br>540-541: It would be strange to talk about mechanisms of effects if in the first place there were no effects or effects are yet to be established. Rather, future studies using larger sample sizes and longer follow up should be undertaken to rigorously establish the effects of DM that may have been missed by this study limited by design and other limitations.<br>203: Exclusion of those with hypoxia might be removing those that probably had the effect of maternal DM. This is a potential bias that should be noted.<br>544-546 Conclusion: The two sentences conflict with each other. They should be revised.

VERSION 2 – AUTHOR RESPONSE

Reviewer: 1
Dr. Dorcas Magai, University of Liverpool
Comments to the Author
Many thanks to the authors for revising the manuscript and for their careful consideration of my previous comments. Please see below additional minor comment:
The last paragraph under the Introduction section would be more appropriately placed under the Limitation section.
Thank you very much for your valuable comments. The paragraph has been moved to the limitations section.

Reviewer: 2
Dr. Margaret Nampijja, African Population and Health Research Center
Comments to the Author
Most of the previous issues have been addressed, and the manuscript has improved. However, there are a few other issues that need to be addressed.
The manuscript has improved; however, a few issues need to be addressed.
Line 158: Exclusion of preterm infants creates a bias since some of the effects of maternal diabetes could be mediated through prematurity. This potential limitation needs to be acknowledged under limitations.
It has been added to the limitations section.
Line 163: Pregnant ladies- women would sound more appropriate.
It has been corrected as suggested.
166: The fasting blood sugar range indicated for diagnosing of DM is incorrect. It should be a fasting glucose result 5.1mmol/L or greater.
It has been corrected as suggested. Thank you very much.
174-175: Provide information on how many children were from in vitro fertilization.
The sentence about in vitro fertilization has been removed as we don’t have the full information about it in this study.
This has been added to the implications and future research as follows:
The method of conception, spontaneous versus in-vitro fertilization, was not investigated in this study, so the impact of that on neurodevelopmental outcomes is a subject for future studies.
State clearly whether any data that was used in this study was primary data or if all data were obtained from records.
It has been clearly stated in the methods section.
Line 197 and 229: Glycated and glycosylated - choose one of the two terms for consistency.
The term Glycosylated has been used all over the manuscript.
Line 200. Clarify what is meant by " No selection of controls was made.'
It has been correction to be “no intentional selection of specific controls has been made”.

Line 234: 'Data analysis and collection were kept in an excel sheet on password secured computers'. The sentence should be revised; does not sound correct.
It has been rephrased and corrected as suggested.
Line 264: The statement " There was no There was no consent for the publication of this research because there was no identity disclosure was made in this study. ' is grammatically not reading right.
It has been corrected as suggested.
Also note that deidentifying data in a publication does not justify a waiver for consenting. In a different place, a different reason was given for not obtaining participant consent. The justification for not having obtained participant consent should be consistent. The key justification here could be the use of secondary data from medical records.
The reason for not obtaining the consent has been unified as suggested. Thank you very much.
Table 1A; The total number N= 5141 should be at the end of the Table heading. In the table itself the statistics in and outside the brackets should be defines e.g. n(%).
It has been corrected as suggested.
439: Provide a description of the MENA region for more context.
It has been written fully as the Middle East and North Africa (MENA).
441: the sentence "....can be attributed to a multifactorial nature”..... sounds incomplete. It should be revised.
It has been revised to be:
These elevated rates of pre-pregnancy diabetes can be due to genetic predispositions, lifestyle factors, better healthcare access, and cultural practices.
443: It is not likely that longitudinal studies can mitigate the incidence of diabetes. This sentence should be revised.
It has been revised to be:
Addressing these factors through further public health initiatives, quality improvement projects and large research studies is crucial to mitigating the rising incidence of diabetes in Qatar.
503: ...higher Apgar scores may have had a protective effect.. this is misleading since as the author later indicates, Apgar score is a reflection of the wellbeing of the newborn. In itself, it is not an exposure. The sentence should be rephrased.
It has been rephrased to be:
Higher Apgar scores, particularly at 5 minutes, are indicative of adequate neonatal adaptation, effective oxygenation, and absence of birth-related hypoxia or asphyxia. The Apgar score is a reflection of the well-being of the newborn. In itself, it is not an exposure.
506: Moreover neurodevelopmental outcomes differed by sex with higher prevalence observed among males. Outcomes could be replaced with impairments.
It has been replaced.
540-541: It would be strange to talk about mechanisms of effects if in the first place there were no effects or effects are yet to be established. Rather, future studies using larger sample sizes and longer follow up should be undertaken to rigorously establish the effects of DM that may have been missed by this study limited by design and other limitations.
It has been rephrased to be:
Despite non-significant associations between gestational diabetes and neurodevelopmental impairments in children in our study, the trends support ongoing surveillance and early screening in children of mothers with gestational diabetes. Future studies with larger sample sizes and longer follow-up should be undertaken to rigorously establish and reaffirm the effects/no effects and associations/no associations of DM on neurodevelopmental outcomes that may have been missed by this study, which was limited by design and other factors.
203: Exclusion of those with hypoxia might be removing those that probably had the effect of maternal DM. This is a potential bias that should be noted.
The following 2 sentences has been added to the limitations as suggested:
Exclusion of those with hypoxia might be removing those who probably had the effect of maternal DM. This is a potential bias that should also be noted.

544-546 Conclusion: The two sentences conflict with each other. They should be revised.
The conclusion has been modified to be:
In conclusion, no significant associations were found between gestational diabetes and early childhood neurodevelopmental delays after adjustment. However, further research studies with a large sample size are needed to assess long-term outcomes in gestational and pre-pregnancy diabetes.

Reviewer: 1
If you have selected ‘Yes’ above, please provide details of any competing interests.: Not applicable

Reviewer: 2
If you have selected ‘Yes’ above, please provide details of any competing interests.: None
